# An Update on Current Pharmacotherapeutic Options for the Treatment of Ulcerative Colitis

**DOI:** 10.3390/jcm11092302

**Published:** 2022-04-20

**Authors:** Francesca Ferretti, Rosanna Cannatelli, Maria Camilla Monico, Giovanni Maconi, Sandro Ardizzone

**Affiliations:** Gastroenterology Unit, ASST Fatebenefratelli-Sacco, Department of Biomedical and Clinical Sciences, “Luigi Sacco” University Hospital, 20157 Milan, Italy; cannatelli.rosanna@asst-fbf-sacco.it (R.C.); monicomariacamilla@gmail.com (M.C.M.); maconi.giovanni@asst-fbf-sacco.it (G.M.); ardizzone.sandro@asst-fbf-sacco.it (S.A.)

**Keywords:** 5-aminosalicylic acid, azathioprine, biologic therapy, corticosteroids, inflammatory bowel disease, small molecule, tofacitinib, ulcerative colitis

## Abstract

The main goals of Ulcerative Colitis (UC) treatment are to both induce and maintain the clinical and endoscopic remission of disease, reduce the incidence of complications such as dysplasia and colorectal carcinoma and improve quality of life. Although a curative medical treatment for UC has not yet been found, new therapeutic strategies addressing specific pathogenetic mechanisms of disease are emerging. Notwithstanding these novel therapies, non-biological conventional drugs remain a mainstay of treatment. The aim of this review is to summarize current therapeutic strategies used as treatment for ulcerative colitis and to briefly focus on emerging therapeutic strategies, including novel biologic therapies and small molecules. To date, multiple therapeutic approaches can be adopted in UC and the range of available compounds is constantly increasing. In this era, the realization of well-designed comparative clinical trials, as well as the definition of specific therapeutic models, would be strongly suggested in order to achieve personalized management for UC patients.

## 1. Introduction

Ulcerative colitis (UC) is a chronic disorder characterized by an inflammatory pattern that usually starts from the rectum and extends proximally in a continuous manner, involving part of or the entire colonic mucosa. UC is an idiopathic disease and although much research is spent on understanding its pathophysiology, the exact aetiology is still unknown. The clinical course of UC is unpredictable, alternating between periods of relapses, characterized by bloody diarrhea and abdominal pain, and periods of remission. The main goals of UC therapy are to both induce and maintain the clinical and endoscopic remission of disease, reduce the incidence of complications and improve quality of life, also avoiding disability [1,2]. UC therapies may be grouped into induction therapies and maintenance therapies. Furthermore, therapeutic strategies are usually guided by the severity (mild, moderate, severe) and extent (proctitis, left sided colitis, pancolitis) of disease. Even if non-biological drugs are the mainstay of treatment, in recent years, the introduction and diffusion of new biologic drugs has emerged as a valid treatment option for UC, also in its earliest stages. At present, despite multiple medical therapies, surgery is the only curative treatment for UC and approximately 15% of UC patients will require proctocolectomy and, if possible, an ileal pouch–anal anastomosis (IPAA) [3,4]. Nonetheless, surgery is associated with a significant morbidity and mortality, as well as a risk of complication of the IPAA; for these reasons, surgery is reserved only to selected cases, such as refractory UC or in the presence of dysplastic or neoplastic lesions [5].

The aim of this review is to summarize current evidence regarding the first- and second-line approach for the management of UC, highlighting the controversial aspects of conventional drugs and the role of emerging strategies, including novel biologic and small molecule therapies.

## 2. Indications for Therapy

According to the most recent guidelines, the management of UC mainly depends on the localization (proctitis, left-sided, extensive) and severity of the disease [6]. Current recommended treatments are summarized in Table 1. The details about the mechanism of action of specific drugs are reported in Table 2, and data about efficacy, dosage, and safety are reported in the following chapters. A specific section is reserved for controversial aspects or open issues about the main approved therapies.

Other factors could influence the therapeutic strategy, such as the disease course and number of relapses, the refractoriness or adverse effects with previous therapies and the onset of extraintestinal manifestations.

Recently, the development of different therapeutic options, such as biologic agents and small molecules, has completely called into question the traditional therapeutic algorithm, and a tailored approach should be recommended.

In particular, a top-down approach is now hypothesized not only for CD but also for UC, even if actual evidence is still scarce [7]. However, a number of long-term consequences can affect UC and should be prevented, bearing high costs in terms of the healthcare system and patients’ quality of life. In particular, among these, we have to mention the need for colectomy in up to 15% of patients [8], a higher risk of colon cancer, extraintestinal manifestations [9] and, even if rarer, also fibrosis and strictures, pseudopolyposis and dysmotility, as well as fecal incontinence [10,11].

For this reason, a personalized approach with a careful assessment of negative prognostic factors and potential risk of under- or over-treatment is suggested in order to avoid negative outcomes and disease progression.

The definition of “high-risk” UC is multifactorial and, according to different studies, can include: young age, male sex, extensive and severe disease activity at the onset, primary sclerosing cholangitis, the need for steroid therapy and non-smoking [12,13,14,15]. Among the abovementioned factors, the endoscopic assessment of severity (i.e., deep ulcerations) is one of the major predictors of future colectomy [14].

**Table 2:** Mechanism of action of Ulcerative Colitis (UC) therapy.

Drug	Action
**Salicylates** [16]	(1) Pro-apoptotic and anti-proliferative action that is triggered, at least in part, by the activation of the peroxisome proliferator-activated receptor (PPAR)-gamma and the modulation of PTEN and c-Myc (2) Have a role in the inhibition of mediators of lipoxygenase and cyclooxygenase, interleukin-1, interleukin-2 and TNF-alpha, and have an antioxidant and free-radical scavenger effect
**Corticosteroids**	Unclear, but it seems to involve the inhibition of cytokine release by inactivation of NFKβ and the consequent reduction in the lymphocyte recruitment, lower vascular permeability and inhibition of cytokine-mediated tissue necrosis
**Calcineurin inhibitors** **Cyclosporine** [17] **Tacrolimus** [17]	Inhibits the activation of T-cells and the production of IL-2 by T-helper lymphocytes, and blocks the production of IFN-xc and B-cell-activating factors A macrolide antibiotic with an anti-calcineurin action similar to CyA
**Thiopurines** **Azathioprine** **Mercapropurine**	Direct anti-inflammatory effect through the inhibition of cytotoxic T-cell and natural killer cells and their apoptosis
**Infliximab** [18]	Binds and blocks both soluble and transmembrane TNF-alpha receptors
**Adalimumab** [19] **Golimumab** [20]	Binds to both soluble and transmembrane-TNF, blocking the reaction with p55 and p 75 subunits of TNF receptors
**Vedolizumab** [21]	Binds the α_4_β_7_ integrin to block the gastrointestinal homing of T lymphocytes, thus reducing the chronic intestinal inflammation present in UC
**Etrolizumab** [22]	Specifically targets the β7 subunit of α4β7 and αEβ7 integrins
**Ustekinumab** [23]	Inhibits the activity of IL-12 and IL-23 by binding to the p40 subunit shared by both cytokines
**Risankizumab**	Binds to the p19 subunit of IL-23
**Mirikizumab**	Binds to the p19 subunit of IL-23
**JAK inhibitors** **Tofacitinib**	Intracellular action on a cascade of multiple pro-inflammatory cytokines

UC, Ulcerative Colitis; PTEN, phosphatase and tensin homolog; CyA, Cyclosporin A; TNF, Tumor Necrosis Factor.

**Figure 1:**
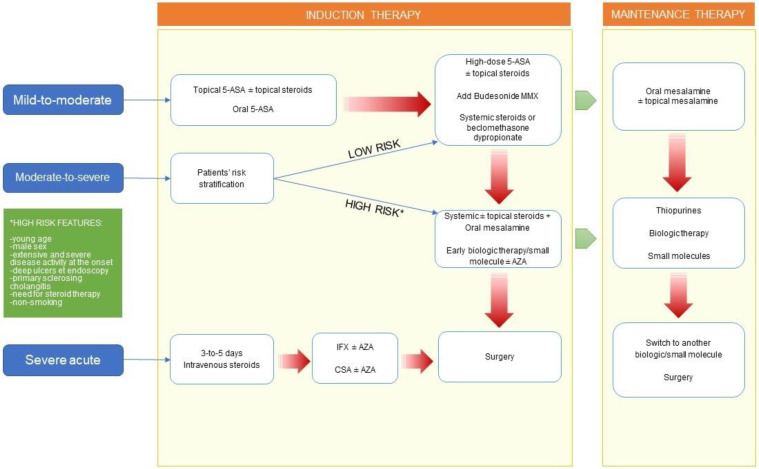
A suggested therapeutic algorithm for Ulcerative Colitis (UC) according to disease severity and patients’ risk stratification. Red arrows indicate non-responders. 5-ASA, mesalamine; MMX, multimatrix; AZA, azathioprine; IFX, infliximab; CSA, cyclosporin.

The rationale is the early initiation of biologic therapies in these patients in order to avoid progression of the disease and its complications. Of course, the main risk is the overtreatment of a mild condition that could be managed with conventional and easy-to-handle drugs. For this reason, the stratification of IBD patients is a necessary step in the decision of the therapeutic algorithm (Figure 1).

## 3. Non-Biologic Therapies

### 3.1. Salicylates

#### 3.1.1. Efficacy

5-aminosalicylic acid (5-ASA) has always had a pivotal and first-line role for both the induction and maintenance of remission in UC [6,24]. In active proctitis, topical mesalamine is the preferred initial treatment [6]. With regard to the formulation, even if mesalamine enemas or foams are possible options, suppositories are usually better tolerated, with a higher efficacy in delivering the drug into the rectum [25].

To date, several formulations are available: in case of proctitis or left-sided colitis, the rectal administration of mesalamine is recommended, whereas oral therapy is suggested in case of more extensive colitis [26]. Moreover, recent oral formulations allow for a selective delivery of the drug in the precise site of inflammation, such as: controlled-release mesalamine for the ileum, the colon and the upper gastrointestinal tract; granulate, delayed-release and multimatrix (MMX) mesalamine acting mainly in the terminal ileum and the colon; and, finally, sulfasalazine (SSZ), balsalazide and olsalazine, which are specifically for the colon [27]. However, the superiority of different mesalamine formulations has not been demonstrated by several studies [28,29].

As demonstrated, the topical application of 5-ASA is more effective than topical steroids [2,30], but the combination between the two compounds is related to better outcomes [31,32]. Moreover, in case of pancolitis or left-sided colitis, the association between oral and topical therapy is the first choice in mild-to-moderate disease [6].

#### 3.1.2. Dosage

According to recent guidelines, the effective oral dosage of salicylates for maintaining remission is at least 2 g/day [2]. The recommended dosage for SSZ is 2 g/day, while higher doses of 5-ASA (>2 g/day) have been demonstrated to give a major benefit compared to patients receiving lower doses [6]. In case of topical rectal treatment, a dosage >1 g/day is recommended in case of relapse; however, the maintenance treatment ranged between 1 g three times per week and 1 g per day [2].

In case of mild disease, the recommended dosage according to the ASCEND I and II trials analysis is 2.4 g/day; however, in case of moderate activity of UC, the initial dose could be higher (up to 4.8 g/day), with a significant benefit for patients [24,33].

Notably, a chemopreventive effect of 5-ASA against colorectal cancer (CRC) has been hypothesized in IBD patients. In particular, a maintenance dose of at least 1.2 g/day relates to a reduced CRC risk [34,35]. On this basis, although a discontinuation of the maintenance therapy could be hypothesized in UC patients on long remission, a long-term treatment could be adopted to lower the CRC risk of IBD patients without contraindications to maintain the drug.

Moreover, SSZ has a well-known effect on rheumatic diseases such as rheumatoid arthritis and polyarticular juvenile idiopathic arthritis. In IBD patients, SSZ is suggested in patients already on remission with SSZ or patients with relevant arthritic symptoms [36].

#### 3.1.3. Safety

Mesalamine preparations are associated with less adverse events (AEs) compared to SSZ, whereas no difference is observed among different mesalamine formulations [37]. The rate of AEs is uncommon (15%); the more frequent ones are some gastrointestinal and extraintestinal effects, such as nausea, abdominal pain, diarrhea and headache; serious reactions are possible but rare, and they include cases of myelotoxicity with leukopenia and agranulocytosis, thrombocytopenia, aplastic anemia, hepatitis, polyarthritis, neurologic manifestations and pericarditis. Thus, a long-term treatment with 5-ASA is generally considered to be safe. The most concerning although rare issue, with a reported incidence of <0.5%, is renal toxicity due to interstitial nephritis; therefore, a periodic monitoring of serum creatinine and urine analysis are recommended [38].

#### 3.1.4. Controversies and Limitations

One of the major limitations of salicylates as maintenance therapy is the degree of adherence to treatment, considering that a low compliance is associated with a worse long-term outcome in terms of recurrent flares, neoplastic complication and, consequently, higher costs for the healthcare system [39,40,41].

In the case of oral mesalamine, the three-times-per-day regimen, the need for multiple pills intake and the fear of side effects are the main factors that determine poor adherence [42,43].

Recently, different studies demonstrated that a simplified regime with a once-daily administration is effective and safe for both clinical and endoscopic remission [37,44], improving the rate of adherence and the patients’ satisfaction also in terms of quality of life [44,45,46].

Moreover, provided that the combination between topical and systemic therapy has proved to be more effective than a single approach [47], the use of suppositories or enemas are associated with a lower adherence than oral therapy [48]. This issue could be overcome with an intermittent regimen of topical therapies. Indeed, in the setting of a chronic disease, a modern management requires a strong patient’s engagement both in the treatment decision and in an adequate education for the correct use of medications.

Moreover, as aforementioned in the Introduction, a gradual evolution from a step-up to a top-down approach is taking hold. Given this, in case of moderate disease at diagnosis, a thorough evaluation of prognostic features should influence the therapeutic approach (Figure 1). In case of high-risk patients, an aggressive approach with systemic corticosteroids or an early use of biologic agents as first-line therapies should be considered rather than a traditional treatment with high-dose salicilates or budesonide-MMX [49].

### 3.2. Corticosteroids

#### 3.2.1. Efficacy

Corticosteroids (CSs) are the recommended therapy in the induction of remission in moderate-to-severe active UC patients and in mild-to-moderate UC patients who do not respond to 5-ASA [6].

CSs can be administered in different formulations: topical (suppositories, foams, enemas) or systemic (oral, intravenous, or intramuscular). In case of active distal colitis, topical steroids can be administered to induce remission, either alone in patients who are intolerant to topical 5-ASA, or in combinations with mesalamine in patients who had an inadequate response to 5-ASA [2,6].

New second-generation CSs have been introduced with a lower bioavailability due to extensive first-pass hepatic metabolism, with reduced systemic side effects, including budesonide and beclomethasone dipropionate (BDP). Budesonide-MMX is a low-systemic absorption CS with a controlled release throughout the colon, making it recommendable for induction therapy in mild-to-moderate UC refractory to mesalamine or in case of intolerance to salicylates [2]. The efficacy and safety of budesonide MMX has been demonstrated by CORE I and II in the induction of clinical and endoscopic remission [50,51]. Moreover, budesonide MMX has also shown its efficacy in the induction phase in a recent systematic review, particularly in left-sided colitis [52]. BDP has demonstrated a similar efficacy compared to oral prednisone in moderate disease [53,54]. In case of severe disease, active UC intravenous therapy is recommended, with a reported efficacy of 67% [55].

#### 3.2.2. Dosage

In case of moderate disease, a course of prednisolone starting from 40 mg/day and tapering 5–10 mg every week is recommended, while a shorter treatment is associated with early relapse [6]. In 2016, a RCT demonstrated that a dose of 5 mg/day of beclomethasone dipropionate (DBP) for 4 weeks and 5 mg/every other day for 4 weeks is not inferior to oral prednisone in mild-to moderate UC [56]. Budesonide MMX should be administered at a dosage of 9 mg day for 8 weeks, without tapering [51,57]. Intravenous therapy is the first-line option in case of severe acute UC since the first study by Truelove and Jewell [58]: to date, a dose of 0.75–1 mg/kg of methylprednisolone or 400 mg of hydrocortisone is recommended [59].

#### 3.2.3. Safety

AEs are quite frequent in patients taking systemic CSs, including weight gain, glycemic disorders, mineral bone loss, an increased risk of infections, psychological disorders and risk of peptic ulcers. To overcome the high incidence of AEs, a second generation of CSs with a limited systemic absorption has been introduced, such as beclomethasone and budesonide. Although not completely absent, these low-bioavailability CSs demonstrate a reduced risk of AEs [60]. A periodic monitoring of blood pressure, lab tests (including complete blood count, blood glucose and serum lipid profile), a mineral bone density assessment and an ophthalmologic evaluation can be suggested in mid-term treatments (>3 months) [61].

#### 3.2.4. Controversies and Limitations

Despite an unquestionable role in the induction of UC remission and in the management of disease flares, CSs are not indicated for maintaining remission due to their AEs, especially in long-term use, including severe cardiovascular events and hip fractures [62,63]. In the last years, low bioavailability CSs, such as budesonide MMX, have been developed to overcome these limitations in mild-to-moderate active UC, and different steroid-sparing drugs (i.e., thiopurines, biologics, small molecules) have appeared on the market. Nevertheless, a corresponding reduction in steroid prescription did not occur [64]. This over-prescription of CSs can probably also be attributed to primary care physicians and medical centers with a lack of expertise in biological drugs and the management of complex IBD. Thus, it is important to stress that a gastroenterologist’s prescription for CS should be at the lowest effective dosage for the shortest necessary period, avoiding incorrect behaviors, such as rapid tapering, too short courses (<3 weeks) and ineffective dosages of prednisolone (<15 mg/day) [6].

Notably, in case of severe ulcerative colitis refractory to steroid therapy, a cytomegalovirus infection should be investigated and treated if present. It is still debated which method is the most accurate to detect an active CMV infection. Multiple bioptic samplings of inflamed colonic tissue (i.e., edges of intestinal ulcers) should be performed and analyzed with immunohistochemistry (IHC) or a polymerase chain reaction (PCR). A definite cut-off has not yet been adopted, even if a viral load of >250 viral copies/mg tissue seems to be acceptable [65]. CMV serology and viraemia are actually considered as optional tests when considering the cessation of immunosuppressive therapy. According to recent guidelines, the best treatment for CMV infection is a dose of 5 mg/kg of intravenous ganciclovir for 5–10 days and a following course of 2–3 weeks of 900 mg/day of valganciclovir [66].

### 3.3. Calcineurin Inhibitors

#### 3.3.1. Cyclosporine

##### Efficacy

CyA has proven its efficacy as a rescue therapy in severe UC. CyA should not be considered as a maintenance therapy but exclusively as a bridge to other medications, such as thiopurines, biologics or surgery [67,68].

##### Dosage

In clinical practice, a lower dose (2 mg/kg/day) is considered the first choice due to the AEs, which are mainly dose-dependent [6].

##### Safety

AEs are mainly dose-dependent and include nephrotoxicity, hypertension, neurotoxicity and a higher risk of infections, including Pneumocystis jirovecii [17].

#### 3.3.2. Tacrolimus

##### Efficacy

According to present guidelines, tacrolimus is suggested as a rescue therapy in acute severe UC and in patients with moderate UC refractory to oral steroids, with a similar effectiveness and safety compared to anti-TNF, as demonstrated in recent studies [69].

##### Dosage

The typical dosage of tacrolimus varies from 0.05 (standard induction) to 0.1 mg/kg/day (rapid induction). Then, the dosage should be adjusted according to a blood concentration of 10–15 ng/mL in the first 2 weeks, and then 5–10 ng/mL. This dosage has demonstrated efficacy in the induction of clinical remission, even if no complete response was obtained [70].

##### Safety

The most common AEs with tacrolimus are neurologic manifestations, such as paresthesia, tremor or headache and renal toxicity [71].

##### Controversies and Limitations

Due to the risk of AEs and despite their efficacy, the role of TAC and CSA is limited to the induction of remission in acute severe steroid–refractory UC or in thiopurines naïve patients with moderate-to-severe UC intolerant or refractory to CS. However, in this setting, the positioning of IFX is debatable and data are often conflicting [72,73]. A recent meta-analysis showed a lower short-term, 1-year and 3-year colectomy rate between IFX and calcineurin inhibitors, whereas no differences were reported in terms of clinical remission. A possible advantage of TAC over CSA can be derived from an indirect comparison, but RCTs are needed to confirm this hypothesis [69].

### 3.4. Immunomodulators

#### 3.4.1. Thiopurines

##### Efficacy

Thiopurines should be considered as a maintenance therapy in patients with mild-to-moderate disease who are intolerant or non-responders to 5-ASA, in patients who are steroid-dependent and in patients responding to cyclosporine or tacrolimus [6]. Due to the delayed effect, thiopurines are not recommended for induction therapy [2]. A meta-analysis of Gisbert et al., including 30 non-RCT (1632 patients), showed a mean efficacy of azathioprine (AZA) of 76% for the maintenance of remission [74]. Moreover, AZA has shown its superiority to salicylates in the maintenance of clinical and endoscopic remission, avoiding the need for steroids, in the management of steroid-dependent UC [75]. Interestingly, based on the comparison between tolerant and intolerant patients to thiopurine, a beneficial effect on the natural history in terms of the cumulative probability of colectomy, hospital admission rates and risk of progression in the disease extent was observed in tolerant patients [76].

Its efficacy can be impaired by excessive methylation via the TPMT route, which can induce an excess of 6-methyl-mercaptopurine (6-MMP) and lower levels of the therapeutically active form, the 6-thioguanine (6-TGN) [77].

##### Dosage

The dosage strictly depends on the patients’ weight. According to available clinical trials, a dosage of 2–3 mg/kg/day has shown a balance between efficacy and adverse effects, whereas, in the case of MP, the equivalent dosage is 1.5 mg/kg/day [78]. Even if randomized controlled trials are lacking, a recent meta-analysis concluded that low-dose AZA (≤1.5 mg/kg/day) could be adequate in chronic active UC patients in terms of efficacy and safety [79]. A once-daily regimen seems to be related to a better adherence to treatment; thus, it is recommended.

##### Safety

Thiopurine methyltransferase (TPMT) and other genetic variants are predisposed to the development of adverse effects, such as myelotoxicity [80,81].

AEs are reported in up to 30% of IBD patients and include dose-dependent toxicities (such as hepatitis and leukopenia) and idiosyncratic reactions (pancreatitis, fever, rash, nausea/vomiting, diarrhea and arthralgias). Thus, it is recommended to closely monitor these possible side effects by screening with a complete blood count and liver function tests every two weeks for the first 2 months, and then, every 3–6 months [82,83].

In case of hepatotoxicity, especially if due to hypermethylation, allopurinol can be added in order to reduce 6-MMP levels, correct hepatotoxicity and enhance the thiopurine response to low-dose AZA [84].

Moreover, a higher incidence of bacterial and viral infections is observed, as well as an increased risk of malignancies. In particular, a higher risk of non-melanoma skin cancer seems to be associated with long-term therapy; the association with other hematological malignancies still remains a controversial topic [85].

##### Controversies and Limitations

Despite their effective role as steroid-sparing agents and the well-known efficacy in maintaining UC remission, AEs are one of the limiting factors for the long-term use of thiopurines. In particular, a definite risk of lymphoma was observed in male patients with a primary Epstein–Barr virus (EBV) infection under 30 or over 50 years old [86]; thus, testing EBV serology before starting therapy is recommended. Moreover, a higher incidence of nonmelanoma skin cancer, including both basal cell and squamous cell carcinoma [87], and of cervical dysplasia, has been reported [88]. Beside neoplastic conditions, both dose-dependent and idiosyncratic adverse reactions can determine the interruption or discontinuation of treatment; thus, periodic monitoring should be performed.

Therefore, considering the current availability of different biologic therapies with good safety profiles, the role of AZA is evolving from monotherapy to combination therapy. In fact, the association between AZA and IFX has proved to be effective in decreasing the risk of the infusion reaction and immunogenicity, preventing the creation of anti-TNF antibodies [89], and a possible role after a previous discontinuation of the monoclonal antibody is under study. In this setting, a recent study demonstrated that a low dose of AZA (1–1.25 mg/kg/day) is comparable to a full dose in achieving therapeutic levels of IFX [90]. At present, the combination therapy with ADA [91], vedolizumab [92] and ustekinumab is still not recommended [23].

## 4. Biologic Therapies

### 4.1. Anti TNF-Alfa

#### 4.1.1. Infliximab (IFX)

##### Efficacy

The role of infliximab (IFX) in the management of moderate-to-severe UC has been reported in two randomized double-blind controlled trials, ACT 1 and 2, including 728 patients overall. IFX was better than the placebo for clinical remission, mucosal healing and steroid sparing [93]. IFX could be use in the induction and maintenance of moderate-to-severe UC patients who failed in conventional therapy [2]. Moreover, the SUCCESS trial demonstrated that the “combo” therapy of IFX and AZA was more effective than monotherapy in the case of moderate-to-severe UC naive to anti-TNF drugs in either corticosteroid-free remission or mucosal healing [94].

IFX has a pivotal role as a rescue therapy, besides intravenous cyclosporine, in acute severe UC inpatients. The efficacy of the two treatments is comparable [95]. However, cyclosporine had a more favorable profile in terms of cost-effectiveness in a recent analysis [96]. In recent years, several biosimilars of the originator infliximab have been developed and widely used. The efficacy and safety of the biosimilars are quite similar to the originator and the switching from the originator to the biosimilar is considered acceptable; whereas multiple switching should be avoided [97]. A good profile of the efficacy and safety of CT-P13 has been reported by a real-life experience of a 2-year follow-up [97,98].

##### Dosage

IFX is administered intravenously, and its maximum concentration is observed approximately one hour after the infusion with a half-life of approximately 14 days [99]. The infusion should last at least two hours [100]; however, a one-hour protocol is adopted in several centers with a good safety profile [101,102,103].

The recommended dosage in UC is 5 mg/kg. The scheduled treatment includes one infusion at week 0, 2 and 6 in the induction phase and one infusion every 8 weeks in the maintenance phase [100]. The ACCENT-1 and ACCENT-2 studies showed twofold higher remission rates with the “every-8-weeks” schedule compared to the placebo groups [104,105,106]. In case of a loss of response, a dose optimization to 10 mg/kg and/or a shortening of the interval between the infusions of up to 4 weeks can be attempted [107]. The cause of a loss of response in up to 40% of patients in clinical remission could be imputable to the development of the antibody to IFX (ATI) [108]. Therefore, the concomitant treatment with immunosuppressants and a scheduled protocol seem to be efficient for reducing the probability in the development of antibodies against the drug. In case of re-treatment after a previous drug withdrawal, a lengthened induction protocol (0, 4 and 8 weeks) has been suggested to reduce the infusion reactions [109]. The pre-treatment with antihistamines, acetaminophen and/or steroid in order to prevent acute infusion reactions and the development of ATI is still controversial.

##### Safety

The most common AE with IFX is related to the infusion. It could be an immediate infusion reaction, which could rise in 5–23% of IBD patients during the infusion until 1–2 h after the infusion, or a late infusion reaction, which could occur in 1–3% of IBD patients 24 h after the infusion. The main symptoms are pruritus, flushing, myalgia and fever. The mild and moderate AE could be treated with graded infusion, slowing the infusion or medical therapy, as well as the use of premedication, for the following infusions. Severe reactions, such as bronchospasm, require epinephrine and the discontinuation of the drug [110].

A recent overview demonstrated that treating UC with biologics in adult populations is a safe approach that is not associated with statistically significant risks of developing serious or opportunistic infections, tuberculosis and malignancies. However, the immunological state of the patient, especially including hepatitis B and C and tuberculosis, should be checked before starting the treatment. In this overview, the total number of malignancies was limited [111]. Notably, patients in treatment with anti-TNF drugs seem to carry a higher risk of cutaneous malignant melanoma; therefore, skin surveillance should be adopted and intervals should be defined by a dermatologist [112].

IFX is considered safe in terms of AEs (AE) and SAEs. However, in a recent meta-analysis including six biological treatments compared with a placebo, only infliximab resulted in a statistically significant worse AE rate (RR = 1.15). However, no statistical relevance was found for SAEs [113].

#### 4.1.2. Adalimumab, Golimumab

##### Efficacy

The role of adalimumab (ADA) in the induction and maintenance of moderate-to-severe UC patients was demonstrated in ULTRA1 and ULTRA 2 trials, with an endpoint of clinical remission at week 8 and 52 [114,115]. The efficacy of golimumab (GOL) in moderate-to-severe UC has been demonstrated by PURSUIT-SC and PURSUIT-M trials [20,116].

The first real-life Italian experience reported that ADA was safe and effective in the induction and maintenance of remission in UC patients [117]. Recently, after the patent expiry of originator ADA, comparable efficacy and safety with biosimilars were demonstrated [97,118].

In a real-life experience, amongst the different anti-TNF treatments, ADA and IFX have shown a similar profile of efficacy for moderate-to-severe UC [119], whereas GOL has shown a lower efficacy, despite a similar good safety profile [120]. Conversely, a recent indirect comparison shows a similar efficacy between IFX and GOL, which were superior to ADA, whereas no difference was observed in the maintenance [121].

##### Dosage

The administration is subcutaneous for both drugs. The recommended dosage of ADA in UC consists of an induction regimen of 160 mg (four 40 mg injections in one day or two 40 mg injections per day in two consecutive days) at week 0, followed by 80 mg 2 weeks later and a maintenance dosage of 40 mg every other week. In case of a loss of response, a dose escalation to 40 mg every week or 80 mg every 2 weeks is recommended [122]. Based on the results of PURSUIT trials [20], the dosage of GOL induction is 200 mg at week 0 and 100 mg at week 2, followed by a dosage of 100 mg every 4 weeks. Notably, in Europe, patients receive 50 or 100 mg according to their body weight being < or >80 Kg, respectively [123]. However, in a recent real-life study, in case of an inadequate response to the induction phase, an early optimization to 100 mg at week 6 has shown a long-term clinical benefit in over half of the patients [124].

##### Safety

ADA was largely used for other immune-mediated diseases and the safety was reported in 23.458 patients who had up to 12 years of clinical exposure. The main adverse event was infection, with an incidence of 1.4/100 patient-years. In patients with pediatric psoriasis, rheumatoid arthritis and Crohn’s diseases, non-melanoma skin cancer incidence was higher compared to the general population, whilst the overall malignancy rates were the same as the general population [125]. The same data were confirmed by a recent analysis including 77 clinical trials [125]. No difference in safety was reported at the every-week dosage [126].

In addition, for GOL, the main adverse event was infections; however, neither opportunistic infection nor tuberculosis were reported. Moreover, increases in the liver enzyme was reported and, out of nine patients, three discontinued the drug [127]. However, for both drugs, the immunological state of the patient should be checked before starting the treatment, as for infliximab. Moreover, injection site reactions, such as erythema and pain, have been reported [115,127].

### 4.2. Anti-Integrins

#### 4.2.1. Vedolizumab

##### Efficacy

The efficacy of induction and maintenance therapy with vedolizumab (VDZ) in UC has been demonstrated in the GEMINI 1 trial, a phase 3, randomized, double-blind, placebo-controlled study in patients with moderate-to-severe UC showing a higher percentage of clinical responses, clinical remission and mucosal healing compared to patients receiving a placebo [92].

Moreover, the VARSITY trial, a double blind, double-dummy, randomized, controlled trial, demonstrated the superiority of VDZ over ADA in patients with moderate-severe UC, both for clinical remission and endoscopic improvement; however, the subgroup analysis did not show the superiority of VDZ over adalimumab in steroid-free clinical remission [128]. Recent guidelines suggested VDZ rather than ADA in the induction and maintenance of moderate-to-severe UC [2,129].

##### Dosage

The recommended dosage of VDZ is 300 mg administered intravenously at weeks 0, 2 and 6, followed by 300 mg intravenously every 8 weeks as maintenance therapy. Nonetheless, several studies have investigated the effect of a dosage intensification to 300 mg every 4 weeks following a secondary loss of response. In particular, a systematic review and meta-analysis by Peyrin-Biroulet et al. demonstrated that dose intensification may be effective in up to 50% of these cases [130]. Furthermore, the subcutaneous route as 108 mg every 2 weeks after intravenous induction has shown a similar efficacy in maintaining remission at week 52 compared to the intravenous route in the VISIBILE study [131].

##### Safety

The long-term safety of VDZ in IBD has been verified by several trials (GEMINI 1, GEMINI 2, GEMINI 3, GEMINI LTS, C13002, C13004), in which, it has been demonstrated that prolonged treatment with VDZ does not increase the risk of AEs and serious AEs compared to the placebo. In particular, no difference in the rate of serious and opportunistic infections has been observed between patients treated with VDZ compared to the placebo [132]. These data have been confirmed by several real-world cohort studies [133,134]. No cases of multifocal leukoencephalopathy in patients treated with VDZ have been reported so far. Furthermore, VDZ has not shown an association with an increased risk of malignancy [135]. In the VISIBILE 1 study, the safety profile of subcutaneous VDZ was similar to that of intravenous VDZ.

### 4.3. Anti-IL23 Agents

#### 4.3.1. Ustekinumab

##### Efficacy

Ustekinumab could be used for induction and maintenance in moderate-to-severe UC failure to respond to conventional therapy [2]. The UNIFI trial, a randomized, double-blind, phase 3, placebo-controlled trial, demonstrated that patients receiving IV ustekinumab at a dose of 130 mg and 6 mg/kg had a significantly higher rate of clinical remission compared to the placebo in the induction and maintenance phase at week 52 [136]. It is important to underline that, in this study, 51% of patients had failed prior biologic therapy and 16.6% of patients had failed both VDZ and anti-TNF.

Until now, “real-life” reports on the efficacy of ustekinumab in UC are very scarce. A recent study has first reported a rate of 53% for ustekinumab achieving clinical remission at 1 year in 19 UC patients refractory or intolerant to all other biologic therapies. In four patients, treatment was stopped due to refractory disease, and one patient discontinued the drug because of a diagnosis of breast cancer after the induction dose. These data are consistent with data reported in the UNIFI trial [137]. More recent studies reported a rate of 32–33% clinical remission at 1 year [138,139].

##### Dosage

Ustekinumab is administered as a single intravenous weight-based induction dose (approximately 6 mg/kg body weight: 55 kg or less, 260 mg; 55 kg to 85 kg, 390 mg; more than 85 kg, 520 mg) followed by a subcutaneous maintenance dosing of 90 mg every 8 weeks or every 12 weeks.

##### Safety

In the UNIFI trial, ustekinumab demonstrated that it had a fairly favorable safety profile. No difference was found in the rates of AEs, serious AEs, infections and serious infections between patients treated with ustekinumab and the placebo, in both the induction and maintenance trial. No opportunistic infections, malignancies or cases of tuberculosis occurred [23].

In a long-term retrospective study, out of 122 CD patients, only five patients stopped ustekinumab, mainly due to infections. Only one serious adverse event (anal adenocarcinoma) was reported in a pediatric onset of CD, after being treated with ustekinumab for 30 months [140].

### 4.4. JAK-Inhibitors

#### 4.4.1. Tofacitinib

Janus kinase (JAK)-inhibitors are a class of small molecules with intracellular action on a cascade of multiple pro-inflammatory cytokines involved in the development of immune-mediated inflammatory diseases, including rheumatoid arthritis and psoriasis.

Recently, the oral formulation of tofacitinib, a JAK1/3 inhibitor [CP-690,550; Pfizer], has been approved for the treatment of moderate-to-severe UC also in Europe [141].

##### Dosage

According to available trials, the induction protocol requires 8 weeks of tofacitinib 10 mg b.i.d., while halving the dose in the maintenance period is suggested in the case of a clinical benefit at week 8. On the contrary, in the case of an inadequate clinical and endoscopic response at week 8, an additional induction period of 8 weeks can be considered. Finally, in the case of an unsatisfactory response after a 16-week treatment, the patient should be considered as a primary non-responder. On the contrary, the maintenance dosage in responder patients is 5 mg b.i.d.

##### Efficacy

The efficacy and safety of tofacitinib on moderate-to-severe UC was demonstrated in phase 2 and phase 3 trials; to date, an open-label, long-term extension trial is ongoing [142,143]. In OCTAVE induction 1 and 2 trials, the primary endpoint was the remission at 8 weeks, and the superiority of tofacitinib (10 mg b.i.d.) versus placebo was demonstrated. An analogous result was observed in terms of mucosal healing. In the maintenance study, a remission rate higher than the placebo was observed both in the 10 mg group and in the 5 mg group. In case of de-escalation (from 10 to 5 mg b.i.d.) after the first 52 weeks, remission was lost in 25.4% of patients [144]. On the contrary, in case of failure of the 5 mg b.i.d., an escalation to 10 mg b.i.d. enabled remission in 49.1% by month 12 [144]. Globally, Sandborn et al. demonstrated that an induction period of 8 to 16 weeks is adequate to induce clinical remission in most patients [145].

##### Safety

Tofacitinib showed an acceptable safety profile. An increased LDL and HDL cholesterol level is a known side effect of tofacitinib, and already reported in trials performed on rheumatologic diseases [146]. However, the clinical meaning of this effect is unknown. Moreover, a dose relationship with herpes zoster infection has been shown, as also demonstrated by a recent meta-analysis [147], even if it did not usually result in permanent treatment discontinuation. Furthermore, HZV vaccination could be adopted at least one month before starting the tofacitinib treatment. In conclusion, the recent trials in UC showed an AE rate that was similar to that of patients on tofacitinib for rheumatoid arthritis or on biologic therapy for UC [148]. Similar to ustekinumab, real-life data for the efficacy and safety of tofacitinib are still poor.

Notably, in July 2019, the US FDA approved a boxed warning applied to the label for an increased risk of pulmonary embolism in patients on 10 mg of tofacitinib b.i.d [149]. Thus, its employment in patients with already known risk factors for venous thromboembolism should be considered with caution. However, Sandborn et al. has recently shown that the safety profile of the induction protocol in UC (10 mg b.i.d) was comparable between the 8-week group and the 16-week group [145].

##### Controversies and Limitations

The Selecting Therapeutic Targets in Inflammatory Bowel Disease (STRIDE) has pointed out the importance of the treat-to-target in IBD. In particular, for UC patients, the target of therapy should be the resolution of clinical symptoms, such as rectal bleeding, and the normalization of bowel habits alongside endoscopic remission, defined as a Mayo endoscopic subscore less than or equal to 1 in the colonoscopy performed 3–6 months after the start of therapy [1].

This study has introduced the concept of “mucosal healing” as a goal of therapy in UC. In fact, a complete mucosal response has been associated with a long-term and steroid-free clinical remission and avoidance of colectomy [150].

A systematic review and meta-analysis explored the efficacy of biologics in the induction and maintenance of mucosal healing in UC. On pooled analysis, both anti-TNF and anti-integrins showed a superiority compared to placebo, inducing remission in 45% of patients versus 30% of patients with the placebo. In particular, infliximab alone or in combination therapy with azathioprine was statistically superior to azathioprine alone in the induction of mucosal healing, with rates of 55%, 63% and 37%, respectively. The superiority of both anti-TNF and anti-integrins over the placebo was confirmed in the maintenance of mucosal healing, with a pooled rate of 33% and 18%, respectively [151].

The importance of achieving mucosal healing in IBD has posed the question of whether the “top-down” approach could lead to better results than the classic “step-up” approach. In the “step-up” approach, patients are treated with a sequential therapy of 5-ASA, steroids, AZA and, finally, biologics; whereas, in the top-down” approach, patients are treated earlier with biologics in order to prevent the progression of disease and bowel damage, such as fibrosis. Although this has a crucial importance in Crohn’s disease, there is less evidence in UC [152].

The early introduction of biological therapy in UC has been explored in several studies [153,154,155,156]. However, no superiority of early treatment with anti-TNF or anti-integrins was found compared to the late start in terms of the rates of colectomy, loss of response and hospitalization [7].

The importance of the stratification of UC patients is increasing in order to tailor the therapy. High-risk factors for UC patients include a young age, male sex, extensive colitis, severe disease activity at diagnosis, primary sclerosing cholangitis, the need for steroids and non-smoking. However, in most studies, the severe activity of disease seems to be the most important negative prognostic factor in the evolution of the disease [12,13,15,157].

Another important point is the loss of efficacy of biologics over time, which happens in 30–50% of patients [107]. This could depend on either the decrease in the trough level of the drug or the production of anti-drug antibodies. The therapeutic drug monitoring (TDM) and the measurement of the anti-drug antibody for anti-TNF are now widely available, and these problems could simply be overcome by either dose optimization or the introduction of an immunosuppressant, respectively [152]. Even if not standardized yet, it seems reasonable to use these tools if they are available in our setting to better drive therapeutic decisions [2,129].

Finally, in several countries, many economic concerns and problems related to the safety and risk of infections and neoplasia have led to the de-escalation and stopping of long-term therapy with biologics in patients in deep and prolonged remission. The European Crohn’s and Colitis Organisation (ECCO) has recently published a topical review about the so-called “exit-strategy” [158]. The concern is that up to 50% of patients develop a relapse at 2 years after stopping biologic therapy and that around 20% will experience a secondary failure to respond to the re-treatment [158]. For this reason, dose escalation and stopping therapy should be tailored in each patient. In particular, good candidates for stopping therapy are patients who did not require dose escalation and those without “high-risk” stigmata.

Obviously, the economic cost of biologics in IBD is still a burden in several countries. Costs related to IBD are derived mostly from drugs, hospitalization and surgery. If it is true that biologics are expensive and the percentage of IBD patients treated with biologics have increased, it is also true that costs related to hospitalization in IBD patients have decreased [159].

Moreover, the advent of biosimilars, which have a more favorable economic impact with an equal efficacy and safety, will have a major influence on IBD-related costs [160].

## 5. Other Therapies

### 5.1. Non-Pharmacological Therapies

Non-pharmacological therapies for UC include probiotics, cytapheresis and fecal transplantation.

Probiotics are living microorganisms that modify and regulate the intestinal microbiota. Even if strong proof is still not available, present evidence suggests a role for probiotics in pouchitis and mild/moderate UC [161]. However, although a positive effect has been suggested in the available trials, both for the induction and maintenance of remission, the optimal approach (dosage, strains, formulations) has not been defined. Among the available preparations, VSL#3 (VSL Pharmaceuticals, Inc., Ft. Lauderdale, FL, a probiotic mix including four strains of Lactobacillus, three strains of Bifidobacterium and one strain of Streptococcus) has recently shown an adequate efficacy in the prevention of the onset of acute pouchitis and relapses in chronic forms; on this basis, the more recent European guidelines suggest its use in the prevention and maintenance of remission in chronic pouchitis [162].

Cytapheresis is a procedure consisting of the selective elimination of productors of inflammatory cytokines, such as activated leucocytes, by an extracorporeal cellular adsorption device. The actual available systems are the granulocyte/monocyte apheresis (GMA) and the leukocytapheresis (LCAP) [163]. Although controversial results are present in available studies, the Adacolumn GMA showed its efficacy in patients with UC; in particular a significant clinical benefit was demonstrated in moderate-to-severe UC and in steroid-dependent patients who failed immunosuppressant and/or biological therapy [164,165]. The LCAP uses the Cellsorba E column and has shown its efficacy in different observational studies, both for the induction of mucosal healing and the improvement of clinical symptoms, even if no-large scale RCTs are available [166,167].

Furthermore, fecal transplantation is emerging as a potential new therapeutic option. However, the efficacy and safety still need to be confirmed [168].

### 5.2. Surgery

The gold standard approach includes total proctocolectomy and the creation of an ileal pouch anal anastomosis.

Up to 10–15% of UC patients will undergo surgical intervention during the natural history of disease; male sex and hospitalization at the diagnosis are among the major risk factors for colectomy [4].

Among therapeutic options, surgery should only be considered as the last resort in refractory severe UC, but also as a timely choice in other situations, such as the risk of neoplastic evolution in patients with specific risk factors [169].

A recent study demonstrated a statistical association between the neoplastic risk and the microscopic inflammation at surveillance endoscopy [170].

With this in mind, it is clear that an adequate approach leading not only to clinical remission but also to a continuous mucosal healing, combining both endoscopic and histologic findings, is a key factor in reducing the need for hospitalization, neoplastic evolution and colectomy rates. Among the available therapies, anti-TNF agents have demonstrated their high efficacy in achieving mucosal healing [171]. Indeed, the incidence of colectomy has decreased since the introduction of biologic agents [172].

### 5.3. Future Perspectives

Different studies on upcoming therapies are still evaluating the efficacy and safety of novel biologic and small molecules for moderate-to-severe UC.

Among biologic agents, etrolizumab is a gut-targeted anti-β7 integrin monoclonal antibody. Phase 1 and 2 trials have been completed for etrolizumab in UC [173]. The phase 2 trial was a randomized, double-blind, placebo-controlled trial to evaluate the efficacy of etrolizumab in moderately to severely active UC, with a subcutaneous dose of etrolizumab of 100 mg at weeks 0, 4 and 8, or 420 mg etrolizumab at week 0 followed by 300 mg at weeks 2, 4 and 8 [174]. The trial showed a significant difference in the number of patients who achieved clinical remission between the group receiving etrolizumab and the placebo. More importantly, the majority (61%) of patients included in this study were non-responders to anti-TNF therapy [174]. AEs occurred with a similar frequency between patients receiving etrolizumab and the placebo. No cases of progressive multifocal leukoencephalopathy were registered [174]. A phase 3 trial has been completed, but the results are still not available [175]. However, etrolizumab failed the primary endpoint for superiority over infliximab in the clinical response at week 10 and clinical remission at week 54 in the phase 3 GARDENIA study [176].

Recently, different interleukin antagonists have been developed and tested.

Risankizumab is a fully human IgG monoclonal antibody inhibitor of IL-23 that is currently approved for the treatment of psoriasis and psoriatic arthritis. For CD, a phase 2 randomized, double-blind, placebo-controlled study with an open label extension has recently been completed, while phase 2 and 3 studies are currently ongoing for UC [177,178]. Regarding the safety, the most common AEs were GI symptoms (nausea, abdominal pain). Otherwise, it was well-tolerated. The rate of serious AEs was not different between patients treated with risankizumab and the placebo (SAEs: risankizumab 200 mg, 22%; risankizumab 600 mg, 7%; placebo, 31%). No other safety issues emerged during the following extended treatment period.

Similarly, mirikizumab is a humanized monoclonal antibody targeting interleukin 23p19 and showed its efficacy in a phase 2 randomized, double-blind, placebo-controlled study on 249 patients (of whom, only one third were naïve) [179]. A dosage of 50 mg, 200 mg and 600 mg every 4 weeks or a placebo was randomly assigned. Significantly higher rates of clinical response at week 12 were reported in the mirikizumab groups compared to the placebo. In the maintenance period, a subcutaneous dose of 200 mg of mirikizumab every 4 or 12 weeks was administered and the achievement of clinical remission at week 52 was 53.7% and 39.7%, respectively. A phase 3 study is still ongoing [180]. Regarding safety, in this study, mirikizumab was well-tolerated. No difference in terms of AEs was found in both groups [179].

In the promising scenario of small molecules, filgotinib, a novel JAK1-preferential inhibitor, demonstrated its efficacy both for the induction and maintenance therapy in the phase 2b/3 SELECTION study [181]. In particular, 37.2% of patients under 200 mg of filgotinib had clinical remission at week 58, and it was statistically significant compared to the placebo group (11.2%).

Upadacitinib, another oral JAK inhibitor that is highly selective for JAK1, showed its superiority to the placebo in terms of efficacy in a phase II UC-ACHIEVE trial [182], while phase III clinical trials are ongoing [183].

Ozanimod, a sphingosine 1-phosphate receptor modulator, was more effective than the placebo for the induction and maintenance of UC patients according to the phase 3 True North study [184] Analogously, etrasimod, an oral S1P receptor-selective modulator, achieved the endpoint of a phase II (OASIS; proof of concept), double-blinded trial, while phase III induction and maintenance studies are currently recruiting [185].

## 6. Conclusions

In conclusion, the treatment of UC can be based on a wide range of therapies that should be decided on the basis of the localization of disease, severity of disease and concomitant conditions. The pivotal role of salicylates has been demonstrated and different formulations are available, both for the induction and maintenance of disease. Moreover, in non-complicated immunosuppressant-naïve patients, treatment with a low dose of 5-ASA should also be continued long-term for its chemo-preventive effect against the development of CRC [35]. Conversely, in patients with an adequate mucosal healing with biologic or small molecule therapies, adding 5-ASA is not necessary, as it has been assessed that intestinal inflammation is a major risk factor in the development of CRC [186].

In case of moderate-to-severe UC patients, CSs are the first-line treatment, and are increasingly more manageable thanks to different available formulations. However, it is necessary to identify corticosteroid-dependent or refractory patients. In case of an adequate response to induction with CSs, AZA should be adopted as a maintenance treatment. On the contrary, in case of a failure to respond to CSs, biological therapies are the first option to evaluate, considering their widespread availability and the growing evidence of efficacy and safety both for the induction and maintenance of remission. Moreover, a “top-down” approach is increasingly adopted, especially in high-risk patients.

Currently, due to the absence of large head-to-head comparative trials, there is no strong evidence to guide the choice of which biologic to administer. To date, the decision should be taken according to patient-specific factors (comorbidities, concomitant extraintestinal manifestations, preferred route of administration), local availability and economic considerations.

Among biologics, anti-TNF agents are usually the first choice due to the great supporting evidence, lower costs and proved efficacy. In acute severe colitis, only infliximab has a demonstrated evidence for such an indication [95]. Moreover, anti-TNF agents should be preferred in the case of concomitant extra-intestinal manifestations (including articular, ocular and dermatologic) of IBD [187].

**Figure 2:**
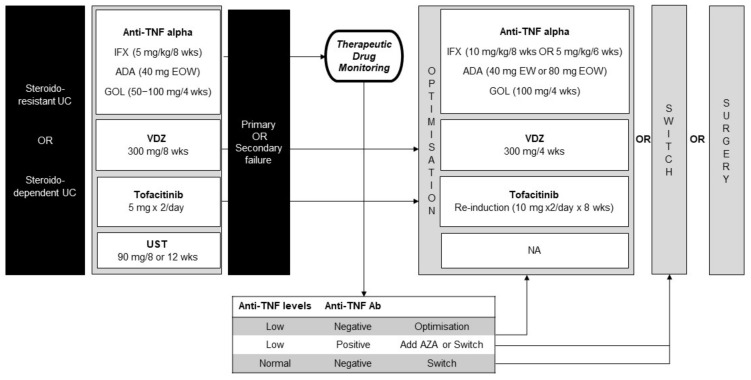
Biologic therapy in the maintenance of remission in UC. UC, ulcerative colitis; IFX, infliximab; wks, weeks; ADA, adalimumab; EOW, every other week; GOL, golimumab; VDZ, vedolizumab; UST, ustekinumab; EW, every week; Ab, antibodies; AZA, azathioprine.

Due to its gut selectivity, VDZ is increasingly being considered as the first choice in the older population or comorbid patients. Recently, ustekinumab has also been approved and could be adopted as a first choice or in the case of a failure to respond to other biologics (Figure 2). Furthermore, growing interest is rising regarding the potential efficacy of small molecules disrupting the intracellular signaling pathways involved in IBD. Among them, tofacitinib has been approved in moderate-to-severe UC and could be adopted as a second- and third-line therapy.

To date, the role of AZA as monotherapy is affected by the availability of new biologic drugs. In fact, the association of AZA and biologics has shown a higher efficacy than monotherapy. In particular, a combination treatment should be evaluated when the efficacy of anti-TNF is lost due to the development of anti-drug antibodies (Figure 2). Moreover, a dual approach with biologics with different mechanism of action is currently under study, mainly in CD, but a hypothesized synergistic effect could also be evaluated in refractory UC.

### Expert Opinion

Salicylates, steroids and immunomodulators are the preferred therapies in up to 60–70% of UC patients. Their application, in terms of indications, efficacy and safety profile, is widely defined by the main national and international guidelines.

Biotechnological drugs and small molecules deserve specific considerations, as an increasing variety of agents are now available. Therefore, physicians will face the complicated decision to choose which drug may be the most suitable for each individual patient.

On one hand, in elderly patients with a history of infectious and neoplastic disease, the choice should be VDZ because of its gut-selective mechanism of action, whereas, for other indications, such as cortico-resistant and cortico-dependent disease or in the presence of extraintestinal manifestations, other biologics should be taken into consideration. However, the choice of which biologic to use is made difficult by the lack of convincing data, especially in controlled trials with a direct comparison between biologics with different mechanisms of action.

Moreover, in our clinical practice, the rules of pharmacoeconomics dictated by regulatory agencies often prevail. This results in a stepwise approach to the use of biologic drugs, usually starting from anti-TNF antagonists and eventually shifting to other biologic therapies after primary or secondary failure. Consequently, recently approved biologic drugs and small molecules will inevitably be used in patients with a history of multiple failures.

Ideally, patients should be stratified in order to identify the most appropriate drug for each individual. In this sense, numerous attempts are underway in the research of various factors (etiological, anamnestic, clinical, laboratory, instrumental, treatment associated or pharmacological), which may direct towards the most suitable treatment option. However, the definition of “personalized” treatment based on therapeutic models is still lacking a valid solution.

Furthermore, it is important to underline that, although several biological agents are currently available for the treatment of UC, response/remission rates still remain unsatisfactory in approximately 60% of cases. A partial explanation could be the limited use of more recent biologic drugs and small molecules in multi-refractory patients, which are known to have a more complicated and long-standing disease. Moreover, to date, a “single-target” therapy is adopted when facing a multifaceted disease with an extremely complex and not completely understood pathophysiology. The objective could be to identify some crucial nodes in the inflammatory process in order to act on key controllers of biological processes thanks to the most advanced bioinformatic tools.

In conclusion, the realization of well-designed clinical trials that comparatively evaluate the efficacy of biologics with different mechanisms of action, as well as the definition of specific therapeutic models through a multitarget approach, will allow, in the future, for the personalization of therapy in patients with UC.

## Figures and Tables

**Table 1:** Recommended first and second-line options in the management of Ulcerative Colitis (UC) according to localization of disease.

	Mild-to-Moderate Disease				Moderate-to-Severe Disease		
Localization	First-Line Options		Second-Line Options		First-Line Options		Refractory
	Induction	Maintenance	Induction	Maintenance	Induction	Maintenance	
**Proctitis**	Topical mesalamine (suppositories/foam) ± combination with topical steroids or oral mesalamine	Oral mesalamine ± topical mesalamine	Systemic steroids Biologics	Immunomodulators Biologics	Systemic steroids ± topical steroids Biologics	Immunomodulators Biologics ± topical mesalamine	Surgery
**Left-sided UC**	Topical mesalamine (enema) ± combination with topical steroids or oral mesalamine Budesonide MMX	Oral mesalamine ± topical mesalamine	Systemic steroids Biologics	Immunomodulators Biologics	Systemic steroids Biologics	Immunomodulators Biologics	Surgery
**Extensive UC**	Topical mesalamine in combination with oral mesalamine Systemic steroids or beclomethasone dipropionate	Oral mesalamine ± topical mesalamine	Biologics	Immunomodulators Biologics	Systemic steroids Biologics	Immunomodulators Biologics	Surgery

UC, Ulcerative Colitis; MMX, Multi Matrix.

## Data Availability

Not applicable.

## References

[1] Peyrin-Biroulet L., Sandborn W., Sands B.E., Reinisch W., Bemelman W., Bryant R.V., D’Haens G., Dotan I., Dubinsky M., Feagan B. (2015). Selecting Therapeutic Targets in Inflammatory Bowel Disease (STRIDE): Determining Therapeutic Goals for Treat-to-Target. Am. J. Gastroenterol..

[2] Raine T., Bonovas S., Burisch J., Kucharzik T., Adamina M., Annese V., Bachmann O., Bettenworth D., Chaparro M., Czuber-Dochan W. (2022). ECCO Guidelines on Therapeutics in Ulcerative Colitis: Medical Treatment. J. Crohn’s Colitis.

[3] Feuerstein J.D., Cheifetz A.S. (2014). Ulcerative colitis: Epidemiology, diagnosis, and management. Mayo Clin. Proc..

[4] Targownik L.E., Singh H., Nugent Z., Bernstein C.N. (2012). The epidemiology of colectomy in ulcerative colitis: Results from a population-based cohort. Am. J. Gastroenterol..

[5] Magro F., Gionchetti P., Eliakim R., Ardizzone S., Armuzzi A., Barreiro-de Acosta M., Burisch J., Gecse K.B., Hart A.L., Hindryckx P. (2017). Third European Evidence-based Consensus on Diagnosis and Management of Ulcerative Colitis. Part 1: Definitions, Diagnosis, Extra-intestinal Manifestations, Pregnancy, Cancer Surveillance, Surgery, and Ileo-anal Pouch Disorders. J. Crohns Colitis.

[6] Harbord M., Eliakim R., Bettenworth D., Karmiris K., Katsanos K., Kopylov U., Kucharzik T., Molnár T., Raine T., Sebastian S. (2017). Third European Evidence-based Consensus on Diagnosis and Management of Ulcerative Colitis. Part 2: Current Management. J. Crohns Colitis.

[7] Berg D.R., Colombel J.F., Ungaro R. (2019). The Role of Early Biologic Therapy in Inflammatory Bowel Disease. Inflamm. Bowel Dis..

[8] Rocchi A., Benchimol E.I., Bernstein C.N., Bitton A., Feagan B., Panaccione R., Glasgow K.W., Fernandes A., Ghosh S. (2012). Inflammatory bowel disease: A Canadian burden of illness review. Can. J. Gastroenterol..

[9] Zallot C., Peyrin-Biroulet L. (2012). Clinical risk factors for complicated disease: How reliable are they?. Dig. Dis..

[10] Gumaste V., Sachar D.B., Greenstein A.J. (1992). Benign and malignant colorectal strictures in ulcerative colitis. Gut.

[11] Torres J., Billioud V., Sachar D.B., Peyrin-Biroulet L., Colombel J.F. (2012). Ulcerative colitis as a progressive disease: The forgotten evidence. Inflamm. Bowel Dis..

[12] Romberg-Camps M.J., Dagnelie P.C., Kester A.D., Hesselink-van de Kruijs M.A., Cilissen M., Engels L.G., Van Deursen C., Hameeteman W.H., Wolters F.L., Russel M.G. (2009). Influence of phenotype at diagnosis and of other potential prognostic factors on the course of inflammatory bowel disease. Am. J. Gastroenterol..

[13] Kuriyama M., Kato J., Fujimoto T., Nasu J., Miyaike J., Morita T., Okada H., Suzuki S., Shiode J., Yamamoto H. (2006). Risk factors and indications for colectomy in ulcerative colitis patients are different according to patient’s clinical background. Dis. Colon Rectum.

[14] Benitez J.M., Louis E. (2014). Can we predict the high-risk patient?. Dig. Dis..

[15] Hefti M.M., Chessin D.B., Harpaz N.H., Steinhagen R.M., Ullman T.A. (2009). Severity of inflammation as a predictor of colectomy in patients with chronic ulcerative colitis. Dis. Colon Rectum.

[16] Iacucci M., de Silva S., Ghosh S. (2010). Mesalazine in inflammatory bowel disease: A trendy topic once again?. Can. J. Gastroenterol..

[17] Loftus C.G., Egan L.J., Sandborn W.J. (2004). Cyclosporine, tacrolimus, and mycophenolate mofetil in the treatment of inflammatory bowel disease. Gastroenterol. Clin. N. Am..

[18] Danese S. (2008). Mechanisms of action of infliximab in inflammatory bowel disease: An anti-inflammatory multitasker. Dig. Liver Dis..

[19] Reinisch W., Sandborn W.J., Hommes D.W., D’Haens G., Hanauer S., Schreiber S., Panaccione R., Fedorak R.N., Tighe M.B., Huang B. (2011). Adalimumab for induction of clinical remission in moderately to severely active ulcerative colitis: Results of a randomised controlled trial. Gut.

[20] Sandborn W.J., Feagan B.G., Marano C., Zhang H., Strauss R., Johanns J., Adedokun O.J., Guzzo C., Colombel J.F., Reinisch W. (2014). Subcutaneous golimumab induces clinical response and remission in patients with moderate-to-severe ulcerative colitis. Gastroenterology.

[21] Wyant T., Fedyk E., Abhyankar B. (2016). An Overview of the Mechanism of Action of the Monoclonal Antibody Vedolizumab. J. Crohns Colitis.

[22] Stefanich E.G., Danilenko D.M., Wang H., O’Byrne S., Erickson R., Gelzleichter T., Hiraragi H., Chiu H., Ivelja S., Jeet S. (2011). A humanized monoclonal antibody targeting the β7 integrin selectively blocks intestinal homing of T lymphocytes. Br. J. Pharmacol..

[23] Feagan B.G., Sandborn W.J., Gasink C., Jacobstein D., Lang Y., Friedman J.R., Blank M.A., Johanns J., Gao L.L., Miao Y. (2016). Ustekinumab as Induction and Maintenance Therapy for Crohn’s Disease. N. Engl. J. Med..

[24] Wang Y., Parker C.E., Feagan B.G., MacDonald J.K. (2016). Oral 5-aminosalicylic acid for maintenance of remission in ulcerative colitis. Cochrane Database Syst. Rev..

[25] Van Bodegraven A.A., Boer R.O., Lourens J., Tuynman H.A., Sindram J.W. (1996). Distribution of mesalazine enemas in active and quiescent ulcerative colitis. Aliment. Pharmacol. Ther..

[26] Marshall J.K., Thabane M., Steinhart A.H., Newman J.R., Anand A., Irvine E.J. (2012). Rectal 5-aminosalicylic acid for maintenance of remission in ulcerative colitis. Cochrane Database Syst. Rev..

[27] Hu M.Y., Peppercorn M.A. (2008). MMX mesalamine: A novel high-dose, once-daily 5-aminosalicylate formulation for the treatment of ulcerative colitis. Expert Opin. Pharmacother..

[28] Levine D.S., Riff D.S., Pruitt R., Wruble L., Koval G., Sales D., Bell J.K., Johnson L.K. (2002). A randomized, double blind, dose-response comparison of balsalazide (6.75 g), balsalazide (2.25 g), and mesalamine (2.4 g) in the treatment of active, mild-to-moderate ulcerative colitis. Am. J. Gastroenterol..

[29] Pruitt R., Hanson J., Safdi M., Wruble L., Hardi R., Johanson J., Koval G., Riff D., Winston B., Cross A. (2002). Balsalazide is superior to mesalamine in the time to improvement of signs and symptoms of acute mild-to-moderate ulcerative colitis. Am. J. Gastroenterol..

[30] Marshall J.K., Irvine E.J. (1997). Rectal corticosteroids versus alternative treatments in ulcerative colitis: A meta-analysis. Gut.

[31] Regueiro M., Loftus E.V., Steinhart A.H., Cohen R.D. (2006). Medical management of left-sided ulcerative colitis and ulcerative proctitis: Critical evaluation of therapeutic trials. Inflamm. Bowel Dis..

[32] Mulder C.J., Fockens P., Meijer J.W., van der Heide H., Wiltink E.H., Tytgat G.N. (1996). Beclomethasone dipropionate (3 mg) versus 5-aminosalicylic acid (2 g) versus the combination of both (3 mg/2 g) as retention enemas in active ulcerative proctitis. Eur. J. Gastroenterol. Hepatol..

[33] Lichtenstein G.R., Ramsey D., Rubin D.T. (2011). Randomised clinical trial: Delayed-release oral mesalazine 4.8 g/day vs. 2.4 g/day in endoscopic mucosal healing—ASCEND I and II combined analysis. Aliment. Pharmacol. Ther..

[34] Qiu X., Ma J., Wang K., Zhang H. (2017). Chemopreventive effects of 5-aminosalicylic acid on inflammatory bowel disease-associated colorectal cancer and dysplasia: A systematic review with meta-analysis. Oncotarget.

[35] Bonovas S., Fiorino G., Lytras T., Nikolopoulos G., Peyrin-Biroulet L., Danese S. (2017). Systematic review with meta-analysis: Use of 5-aminosalicylates and risk of colorectal neoplasia in patients with inflammatory bowel disease. Aliment. Pharmacol. Ther..

[36] Ko C.W., Singh S., Feuerstein J.D., Falck-Ytter C., Falck-Ytter Y., Cross R.K., on behalf of the American Gastroenterological Association Institute Clinical Guidelines Committee (2019). AGA Clinical Practice Guidelines on the Management of Mild-to-Moderate Ulcerative Colitis. Gastroenterology.

[37] Murray A., Nguyen T.M., Parker C.E., Feagan B.G., MacDonald J.K. (2020). Oral 5-aminosalicylic acid for maintenance of remission in ulcerative colitis. Cochrane Database Syst. Rev..

[38] Gisbert J.P., González-Lama Y., Maté J. (2007). 5-Aminosalicylates and renal function in inflammatory bowel disease: A systematic review. Inflamm. Bowel Dis..

[39] Kane S., Huo D., Aikens J., Hanauer S. (2003). Medication nonadherence and the outcomes of patients with quiescent ulcerative colitis. Am. J. Med..

[40] Moody G.A., Jayanthi V., Probert C.S., Mac Kay H., Mayberry J.F. (1996). Long-term therapy with sulphasalazine protects against colorectal cancer in ulcerative colitis: A retrospective study of colorectal cancer risk and compliance with treatment in Leicestershire. Eur. J. Gastroenterol. Hepatol..

[41] Mitra D., Hodgkins P., Yen L., Davis K.L., Cohen R.D. (2012). Association between oral 5-ASA adherence and health care utilization and costs among patients with active ulcerative colitis. BMC Gastroenterol..

[42] Shale M.J., Riley S.A. (2003). Studies of compliance with delayed-release mesalazine therapy in patients with inflammatory bowel disease. Aliment. Pharmacol. Ther..

[43] Kane S.V. (2006). Systematic review: Adherence issues in the treatment of ulcerative colitis. Aliment. Pharmacol. Ther..

[44] D’Haens G.R., Sandborn W.J., Zou G., Stitt L.W., Rutgeerts P.J., Gilgen D., Jairath V., Hindryckx P., Shackelton L.M., Vandervoort M.K. (2017). Randomised non-inferiority trial: 1600 mg versus 400 mg tablets of mesalazine for the treatment of mild-to-moderate ulcerative colitis. Aliment. Pharmacol. Ther..

[45] Flourié B., Hagège H., Tucat G., Maetz D., Hébuterne X., Kuyvenhoven J.P., Tan T.G., Pierik M.J., Masclee A.A., Dewit O. (2013). Randomised clinical trial: Once- vs. twice-daily prolonged-release mesalazine for active ulcerative colitis. Aliment. Pharmacol. Ther..

[46] Sandborn W.J., Korzenik J., Lashner B., Leighton J.A., Mahadevan U., Marion J.F., Safdi M., Sninsky C.A., Patel R.M., Friedenberg K.A. (2010). Once-daily dosing of delayed-release oral mesalamine (400-mg tablet) is as effective as twice-daily dosing for maintenance of remission of ulcerative colitis. Gastroenterology.

[47] Singh S., Feuerstein J.D., Binion D.G., Tremaine W.J. (2019). AGA Technical Review on the Management of Mild-to-Moderate Ulcerative Colitis. Gastroenterology.

[48] D’Incà R., Bertomoro P., Mazzocco K., Vettorato M.G., Rumiati R., Sturniolo G.C. (2008). Risk factors for non-adherence to medication in inflammatory bowel disease patients. Aliment. Pharmacol. Ther..

[49] Dassopoulos T., Cohen R.D., Scherl E.J., Schwartz R.M., Kosinski L., Regueiro M.D. (2015). Ulcerative Colitis Care Pathway. Gastroenterology.

[50] Sandborn W.J., Travis S., Moro L., Jones R., Gautille T., Bagin R., Huang M., Yeung P., Ballard E.D. (2012). Once-daily budesonide MMX^®^ extended-release tablets induce remission in patients with mild to moderate ulcerative colitis: Results from the CORE I study. Gastroenterology.

[51] Travis S.P., Danese S., Kupcinskas L., Alexeeva O., D’Haens G., Gibson P.R., Moro L., Jones R., Ballard E.D., Masure J. (2014). Once-daily budesonide MMX in active, mild-to-moderate ulcerative colitis: Results from the randomised CORE II study. Gut.

[52] Sherlock M.E., MacDonald J.K., Griffiths A.M., Steinhart A.H., Seow C.H. (2015). Oral budesonide for induction of remission in ulcerative colitis. Cochrane Database Syst. Rev..

[53] Rizzello F., Gionchetti P., D’Arienzo A., Manguso F., Di Matteo G., Annese V., Valpiani D., Casetti T., Adamo S., Prada A. (2002). Oral beclometasone dipropionate in the treatment of active ulcerative colitis: A double-blind placebo-controlled study. Aliment. Pharmacol. Ther..

[54] Campieri M., Adamo S., Valpiani D., D’Arienzo A., D’Albasio G., Pitzalis M., Cesari P., Casetti T., Castiglione G.N., Rizzello F. (2003). Oral beclometasone dipropionate in the treatment of extensive and left-sided active ulcerative colitis: A multicentre randomised study. Aliment. Pharmacol. Ther..

[55] Turner D., Walsh C.M., Steinhart A.H., Griffiths A.M. (2007). Response to corticosteroids in severe ulcerative colitis: A systematic review of the literature and a meta-regression. Clin. Gastroenterol. Hepatol..

[56] Van Assche G., Manguso F., Zibellini M., Nuño J.L.C., Goldis A., Tkachenko E., Varoli G., Kleczkowski D., Annese V., D’Heygere F. (2015). Oral prolonged release beclomethasone dipropionate and prednisone in the treatment of active ulcerative colitis: Results from a double-blind, randomized, parallel group study. Am. J. Gastroenterol..

[57] Rubin D.T., Cohen R.D., Sandborn W.J., Lichtenstein G.R., Axler J., Riddell R.H., Zhu C., Barrett A.C., Bortey E., Forbes W.P. (2017). Budesonide Multimatrix Is Efficacious for Mesalamine-refractory, Mild to Moderate Ulcerative Colitis: A Randomised, Placebo-controlled Trial. J. Crohns Colitis.

[58] Truelove S.C., Jewell D.P. (1974). Intensive intravenous regimen for severe attacks of ulcerative colitis. Lancet.

[59] Gionchetti P., Rizzello F., Annese V., Armuzzi A., Biancone L., Castiglione F., Comberlato M., Cottone M., Danese S., Daperno M. (2017). Use of corticosteroids and immunosuppressive drugs in inflammatory bowel disease: Clinical practice guidelines of the Italian Group for the Study of Inflammatory Bowel Disease. Dig. Liver Dis..

[60] De Cassan C., Fiorino G., Danese S. (2012). Second-generation corticosteroids for the treatment of Crohn’s disease and ulcerative colitis: More effective and less side effects?. Dig. Dis..

[61] Liu D., Ahmet A., Ward L., Krishnamoorthy P., Mandelcorn E.D., Leigh R., Brown J.P., Cohen A., Kim H. (2013). A practical guide to the monitoring and management of the complications of systemic corticosteroid therapy. Allergy Asthma Clin. Immunol..

[62] Lewis J.D., Scott F.I., Brensinger C.M., Roy J.A., Osterman M.T., Mamtani R., Bewtra M., Chen L., Yun H., Xie F. (2018). Increased Mortality Rates with Prolonged Corticosteroid Therapy When Compared with Antitumor Necrosis Factor-α-Directed Therapy for Inflammatory Bowel Disease. Am. J. Gastroenterol..

[63] Selinger C.P., Parkes G.C., Bassi A., Limdi J.K., Ludlow H., Patel P., Smith M., Saluke S., Ndlovu Z., George B. (2019). Assessment of steroid use as a key performance indicator in inflammatory bowel disease-analysis of data from 2385 UK patients. Aliment. Pharmacol. Ther..

[64] Chhaya V., Saxena S., Cecil E., Subramanian V., Curcin V., Majeed A., Pollok R.C. (2016). Steroid dependency and trends in prescribing for inflammatory bowel disease—A 20-year national population-based study. Aliment. Pharmacol. Ther..

[65] Roblin X., Pillet S., Oussalah A., Berthelot P., Del Tedesco E., Phelip J.M., Chambonniere M.L., Garraud O., Peyrin-Biroulet L., Pozzetto B. (2011). Cytomegalovirus load in inflamed intestinal tissue is predictive of resistance to immunosuppressive therapy in ulcerative colitis. Am. J. Gastroenterol..

[66] Kucharzik T., Ellul P., Greuter T., Rahier J.F., Verstockt B., Abreu C., Albuquerque A., Allocca M., Esteve M., Farraye F.A. (2021). ECCO Guidelines on the Prevention, Diagnosis, and Management of Infections in Inflammatory Bowel Disease. J. Crohn’s Colitis.

[67] Lichtiger S., Present D.H., Kornbluth A., Gelernt I., Bauer J., Galler G., Michelassi F., Hanauer S. (1994). Cyclosporine in severe ulcerative colitis refractory to steroid therapy. N. Engl. J. Med..

[68] D’Haens G., Lemmens L., Geboes K., Vandeputte L., Van Acker F., Mortelmans L., Peeters M., Vermeire S., Penninckx F., Nevens F. (2001). Intravenous cyclosporine versus intravenous corticosteroids as single therapy for severe attacks of ulcerative colitis. Gastroenterology.

[69] Jia X., Guo R., Hu Z., Liu J., Li B., Yang Q., He J. (2020). Efficacy of infliximab, cyclosporine and tacrolimus on ulcerative colitis: A meta-analysis. Medicine.

[70] Ogata H., Matsui T., Nakamura M., Iida M., Takazoe M., Suzuki Y., Hibi T. (2006). A randomised dose finding study of oral tacrolimus (FK506) therapy in refractory ulcerative colitis. Gut.

[71] Baumgart D.C., Pintoffl J.P., Sturm A., Wiedenmann B., Dignass A.U. (2006). Tacrolimus is safe and effective in patients with severe steroid-refractory or steroid-dependent inflammatory bowel disease—A long-term follow-up. Am. J. Gastroenterol..

[72] Sjöberg M., Walch A., Meshkat M., Gustavsson A., Järnerot G., Vogelsang H., Hertervig E., Novacek G., Friis-Liby I., Blomquist L. (2012). Infliximab or cyclosporine as rescue therapy in hospitalized patients with steroid-refractory ulcerative colitis: A retrospective observational study. Inflamm. Bowel Dis..

[73] Croft A., Walsh A., Doecke J., Cooley R., Howlett M., Radford-Smith G. (2013). Outcomes of salvage therapy for steroid-refractory acute severe ulcerative colitis: Ciclosporin vs. infliximab. Aliment. Pharmacol. Ther..

[74] Gisbert J.P., Linares P.M., McNicholl A.G., Maté J., Gomollón F. (2009). Meta-analysis: The efficacy of azathioprine and mercaptopurine in ulcerative colitis. Aliment. Pharmacol. Ther..

[75] Ardizzone S., Maconi G., Russo A., Imbesi V., Colombo E., Bianchi Porro G. (2006). Randomised controlled trial of azathioprine and 5-aminosalicylic acid for treatment of steroid dependent ulcerative colitis. Gut.

[76] Eriksson C., Rundquist S., Cao Y., Montgomery S., Halfvarson J. (2019). Impact of thiopurines on the natural history and surgical outcome of ulcerative colitis: A cohort study. Gut.

[77] Dubinsky M.C., Yang H., Hassard P.V., Seidman E.G., Kam L.Y., Abreu M.T., Targan S.R., Vasiliauskas E.A. (2002). 6-MP metabolite profiles provide a biochemical explanation for 6-MP resistance in patients with inflammatory bowel disease. Gastroenterology.

[78] Prefontaine E., Sutherland L.R., Macdonald J.K., Cepoiu M. (2009). Azathioprine or 6-mercaptopurine for maintenance of remission in Crohn’s disease. Cochrane Database Syst. Rev..

[79] Luan Z.J., Li Y., Zhao X.Y., Wang L., Sun Y.H., Wang S.Y., Qian J.M. (2016). Treatment efficacy and safety of low-dose azathioprine in chronic active ulcerative colitis patients: A meta-analysis and systemic review. J. Dig. Dis..

[80] Yang S.K., Hong M., Baek J., Choi H., Zhao W., Jung Y., Haritunians T., Ye B.D., Kim K.J., Park S.H. (2014). A common missense variant in NUDT15 confers susceptibility to thiopurine-induced leukopenia. Nat. Genet..

[81] Zabala W., Cruz R., Barreiro-de Acosta M., Chaparro M., Panes J., Echarri A., Esteve M., Carpio D., Andreu M., García-Planella E. (2013). New genetic associations in thiopurine-related bone marrow toxicity among inflammatory bowel disease patients. Pharmacogenomics.

[82] Chaparro M., Ordás I., Cabré E., Garcia-Sanchez V., Bastida G., Peñalva M., Gomollón F., García-Planella E., Merino O., Gutiérrez A. (2013). Safety of thiopurine therapy in inflammatory bowel disease: Long-term follow-up study of 3931 patients. Inflamm. Bowel Dis..

[83] Gisbert J.P., González-Lama Y., Maté J. (2007). Thiopurine-induced liver injury in patients with inflammatory bowel disease: A systematic review. Am. J. Gastroenterol..

[84] Moreau B., Clement P., Theoret Y., Seidman E.G. (2017). Allopurinol in combination with thiopurine induces mucosal healing and improves clinical and metabolic outcomes in IBD. Ther. Adv. Gastroenterol..

[85] Kotlyar D.S., Lewis J.D., Beaugerie L., Tierney A., Brensinger C.M., Gisbert J.P., Loftus E.V., Peyrin-Biroulet L., Blonski W.C., Van Domselaar M. (2015). Risk of lymphoma in patients with inflammatory bowel disease treated with azathioprine and 6-mercaptopurine: A meta-analysis. Clin. Gastroenterol. Hepatol..

[86] Dayharsh G.A., Loftus E.V., Sandborn W.J., Tremaine W.J., Zinsmeister A.R., Witzig T.E., Macon W.R., Burgart L.J. (2002). Epstein-Barr virus-positive lymphoma in patients with inflammatory bowel disease treated with azathioprine or 6-mercaptopurine. Gastroenterology.

[87] Singh H., Nugent Z., Demers A.A., Bernstein C.N. (2011). Increased risk of nonmelanoma skin cancers among individuals with inflammatory bowel disease. Gastroenterology.

[88] Hazenberg H.M.J.L., de Boer N.K.H., Mulder C.J.J., Mom S.H., van Bodegraven A.A., Tack G.J. (2018). Neoplasia and Precursor Lesions of the Female Genital Tract in IBD: Epidemiology, Role of Immunosuppressants, and Clinical Implications. Inflamm. Bowel Dis..

[89] Mogensen D.V., Brynskov J., Ainsworth M.A., Nersting J., Schmiegelow K., Steenholdt C. (2018). A Role for Thiopurine Metabolites in the Synergism Between Thiopurines and Infliximab in Inflammatory Bowel Disease. J. Crohns Colitis.

[90] Yarur A.J., Kubiliun M.J., Czul F., Sussman D.A., Quintero M.A., Jain A., Drake K.A., Hauenstein S.I., Lockton S., Deshpande A.R. (2015). Concentrations of 6-thioguanine nucleotide correlate with trough levels of infliximab in patients with inflammatory bowel disease on combination therapy. Clin. Gastroenterol. Hepatol..

[91] Chalhoub J.M., Rimmani H.H., Gumaste V.V., Sharara A.I. (2017). Systematic Review and Meta-analysis: Adalimumab Monotherapy Versus Combination Therapy with Immunomodulators for Induction and Maintenance of Remission and Response in Patients with Crohn’s Disease. Inflamm. Bowel Dis..

[92] Feagan B.G., Rutgeerts P., Sands B.E., Hanauer S., Colombel J.F., Sandborn W.J., Van Assche G., Axler J., Kim H.J., Danese S. (2013). Vedolizumab as induction and maintenance therapy for ulcerative colitis. N. Engl. J. Med..

[93] Rutgeerts P., Sandborn W.J., Feagan B.G., Reinisch W., Olson A., Johanns J., Travers S., Rachmilewitz D., Hanauer S.B., Lichtenstein G.R. (2005). Infliximab for induction and maintenance therapy for ulcerative colitis. N. Engl. J. Med..

[94] Panaccione R., Ghosh S., Middleton S., Márquez J.R., Scott B.B., Flint L., van Hoogstraten H.J., Chen A.C., Zheng H., Danese S. (2014). Combination therapy with infliximab and azathioprine is superior to monotherapy with either agent in ulcerative colitis. Gastroenterology.

[95] Laharie D., Bourreille A., Branche J., Allez M., Bouhnik Y., Filippi J., Zerbib F., Savoye G., Nachury M., Moreau J. (2012). Ciclosporin versus infliximab in patients with severe ulcerative colitis refractory to intravenous steroids: A parallel, open-label randomised controlled trial. Lancet.

[96] Seagrove A.C., Alam M.F., Alrubaiy L., Cheung W.Y., Clement C., Cohen D., Grey M., Hilton M., Hutchings H., Morgan J. (2014). Randomised controlled trial. Comparison Of iNfliximab and ciclosporin in STeroid Resistant Ulcerative Colitis: Trial design and protocol (CONSTRUCT). BMJ Open.

[97] Fiorino G., Caprioli F., Daperno M., Mocciaro F., Principi M., Viscido A., Fantini M.C., Orlando A., Papi C., Annese V. (2019). Use of biosimilars in inflammatory bowel disease: A position update of the Italian Group for the Study of Inflammatory Bowel Disease (IG-IBD). Dig. Liver Dis..

[98] Guerra Veloz M.F., Belvis Jiménez M., Valdes Delgado T., Castro Laria L., Maldonado Pérez B., Perea Amarillo R., Merino Bohórquez V., Caunedo Álvarez Á., Vilches Arenas Á., Argüelles-Arias F. (2019). Long-term follow up after switching from original infliximab to an infliximab biosimilar: Real-world data. Ther. Adv. Gastroenterol..

[99] Hemperly A., Vande Casteele N. (2018). Clinical Pharmacokinetics and Pharmacodynamics of Infliximab in the Treatment of Inflammatory Bowel Disease. Clin. Pharmacokinet..

[100] (2013). REMICADE, ^®^ (Infliximab) [Package Insert].

[101] Michielan A., Martinato M., Favarin A., Zanotto V., Caccaro R., Caruso A., Sturniolo G.C., D’Incà R. (2015). A nurse-led accelerated procedure for infliximab infusion is well tolerated and effective in patients with inflammatory bowel disease. Dig. Liver Dis..

[102] Babouri A., Roblin X., Filippi J., Hébuterne X., Bigard M.A., Peyrin-Biroulet L. (2014). Tolerability of one hour 10mg/kg infliximab infusions in inflammatory bowel diseases: A prospective multicenter cohort study. J. Crohns Colitis.

[103] Lee T.W., Singh R., Fedorak R.N. (2011). A one-hour infusion of infliximab during maintenance therapy is safe and well tolerated: A prospective cohort study. Aliment. Pharmacol. Ther..

[104] Hanauer S.B., Feagan B.G., Lichtenstein G.R., Mayer L.F., Schreiber S., Colombel J.F., Rachmilewitz D., Wolf D.C., Olson A., Bao W. (2002). Maintenance infliximab for Crohn’s disease: The ACCENT I randomised trial. Lancet.

[105] Sands B.E., Anderson F.H., Bernstein C.N., Chey W.Y., Feagan B.G., Fedorak R.N., Kamm M.A., Korzenik J.R., Lashner B.A., Onken J.E. (2004). Infliximab maintenance therapy for fistulizing Crohn’s disease. N. Engl. J. Med..

[106] Sandborn W.J., Rutgeerts P., Feagan B.G., Reinisch W., Olson A., Johanns J., Lu J., Horgan K., Rachmilewitz D., Hanauer S.B. (2009). Colectomy rate comparison after treatment of ulcerative colitis with placebo or infliximab. Gastroenterology.

[107] Ben-Horin S., Chowers Y. (2011). Review article: Loss of response to anti-TNF treatments in Crohn’s disease. Aliment. Pharmacol. Ther..

[108] Chaparro M., Guerra I., Muñoz-Linares P., Gisbert J.P. (2012). Systematic review: Antibodies and anti-TNF-α levels in inflammatory bowel disease. Aliment. Pharmacol. Ther..

[109] Iannone L.F., Bennardo L., Palleria C., Roberti R., De Sarro C., Naturale M.D., Dastoli S., Donato L., Manti A., Valenti G. (2020). Safety profile of biologic drugs for psoriasis in clinical practice: An Italian prospective pharmacovigilance study. PLoS ONE.

[110] Lichtenstein L., Ron Y., Kivity S., Ben-Horin S., Israeli E., Fraser G.M., Dotan I., Chowers Y., Confino-Cohen R., Weiss B. (2015). Infliximab-Related Infusion Reactions: Systematic Review. J. Crohn’s Colitis.

[111] Bonovas S., Pantavou K., Evripidou D., Bastiampillai A.J., Nikolopoulos G.K., Peyrin-Biroulet L., Danese S. (2018). Safety of biological therapies in ulcerative colitis: An umbrella review of meta-analyses. Best Pract. Res. Clin. Gastroenterol..

[112] Annese V., Beaugerie L., Egan L., Biancone L., Bolling C., Brandts C., Dierickx D., Dummer R., Fiorino G., Gornet J.M. (2015). European Evidence-based Consensus: Inflammatory Bowel Disease and Malignancies. J. Crohn’s Colitis.

[113] Zhou H.Y., Guo B., Lufumpa E., Li X.M., Chen L.H., Meng X., Li B.Z. (2020). Comparative of the Effectiveness and Safety of Biological Agents, Tofacitinib, and Fecal Microbiota Transplantation in Ulcerative Colitis: Systematic Review and Network Meta-Analysis. Immunol. Investig..

[114] Sandborn W., Van Assche G., Reinisch W. (2013). Adalimumab in the Treatment of Moderate-to-Severe Ulcerative Colitis: ULTRA 2 Trial Results. Gastroenterol. Hepatol. (NY).

[115] Colombel J.F., Sandborn W.J., Ghosh S., Wolf D.C., Panaccione R., Feagan B., Reinisch W., Robinson A.M., Lazar A., Kron M. (2014). Four-year maintenance treatment with adalimumab in patients with moderately to severely active ulcerative colitis: Data from ULTRA 1, 2, and 3. Am. J. Gastroenterol..

[116] Sandborn W.J., Feagan B.G., Marano C., Zhang H., Strauss R., Johanns J., Adedokun O.J., Guzzo C., Colombel J.F., Reinisch W. (2014). Subcutaneous golimumab maintains clinical response in patients with moderate-to-severe ulcerative colitis. Gastroenterology.

[117] Tursi A., Elisei W., Faggiani R., Allegretta L., Valle N.D., Forti G., Franceschi M., Ferronato A., Gallina S., Larussa T. (2018). Effectiveness and safety of adalimumab to treat outpatient ulcerative colitis: A real-life multicenter, observational study in primary inflammatory bowel disease centers. Medicine.

[118] Lukas M., Malickova K., Kolar M., Bortlik M., Vasatko M., Machkova N., Hruba V., Duricova D. (2020). Switching from Originator Adalimumab to the Biosimilar SB5 in Patients with Inflammatory Bowel Disease: Short-term Experience from a Single Tertiary Clinical Centre. J. Crohns Colitis.

[119] Sandborn W.J., Sakuraba A., Wang A., Macaulay D., Reichmann W., Wang S., Chao J., Skup M. (2016). Comparison of real-world outcomes of adalimumab and infliximab for patients with ulcerative colitis in the United States. Curr. Med. Res. Opin..

[120] Barberio B., Zingone F., Frazzoni L., D’Incà R., Maccarone M.C., Ghisa M., Massimi D., Lorenzon G., Savarino E. (2020). Real life comparison of different anti-TNF biologic therapies for ulcerative colitis treatment: A retrospective cohort study. Dig. Dis..

[121] Kawalec P., Pilc A. (2016). An indirect comparison of infliximab versus adalimumab or golimumab for active ulcerative colitis. Arch. Med. Sci..

[122] (2015). HUMIRA, ^®^ (Adalimumab) [Package Insert].

[123] (2015). SIMPONI, ^®^ (Golimumab) [Package Insert].

[124] Taxonera C., Iborra M., Bosca-Watts M.M., Rubio S., Nantes Ó., Higuera R., Bertoletti F., Martínez-Montiel P., Sierra-Ausin M., Manceñido N. (2019). Early dose optimization of golimumab induces late response and long-term clinical benefit in moderately to severely active ulcerative colitis. Curr. Med. Res. Opin..

[125] Burmester G.R., Gordon K.B., Rosenbaum J.T., Arikan D., Lau W.L., Li P., Faccin F., Panaccione R. (2020). Long-Term Safety of Adalimumab in 29,967 Adult Patients from Global Clinical Trials Across Multiple Indications: An Updated Analysis. Adv. Ther..

[126] Ryan C., Sobell J.M., Leonardi C.L., Lynde C.W., Karunaratne M., Valdecantos W.C., Hendrickson B.A. (2018). Safety of Adalimumab Dosed Every Week and Every Other Week: Focus on Patients with Hidradenitis Suppurativa or Psoriasis. Am. J. Clin. Dermatol..

[127] Inman R.D., Davis J.C., Heijde D., Diekman L., Sieper J., Kim S.I., Mack M., Han J., Visvanathan S., Xu Z. (2008). Efficacy and safety of golimumab in patients with ankylosing spondylitis: Results of a randomized, double-blind, placebo-controlled, phase III trial. Arthritis Rheum..

[128] Sands B.E., Peyrin-Biroulet L., Loftus E.V., Danese S., Colombel J.F., Törüner M., Jonaitis L., Abhyankar B., Chen J., Rogers R. (2019). Vedolizumab versus Adalimumab for Moderate-to-Severe Ulcerative Colitis. N. Engl. J. Med..

[129] Macaluso F.S., Orlando A., Papi C., Festa S., Pugliese D., Bonovas S., Pansieri C., Piovani D., Fiorino G., Fantini M.C. (2022). Use of biologics and small molecule drugs for the management of moderate to severe ulcerative colitis: IG-IBD clinical guidelines based on the GRADE methodology. Dig. Liver Dis..

[130] Peyrin-Biroulet L., Danese S., Argollo M., Pouillon L., Peppas S., Gonzalez-Lorenzo M., Lytras T., Bonovas S. (2019). Loss of Response to Vedolizumab and Ability of Dose Intensification to Restore Response in Patients with Crohn’s Disease or Ulcerative Colitis: A Systematic Review and Meta-analysis. Clin. Gastroenterol. Hepatol..

[131] Sandborn W.J., Baert F., Danese S., Krznarić Ž., Kobayashi T., Yao X., Chen J., Rosario M., Bhatia S., Kisfalvi K. (2020). Efficacy and Safety of Vedolizumab Subcutaneous Formulation in a Randomized Trial of Patients with Ulcerative Colitis. Gastroenterology.

[132] Colombel J.F., Sands B.E., Rutgeerts P., Sandborn W., Danese S., D’Haens G., Panaccione R., Loftus E.V., Sankoh S., Fox I. (2017). The safety of vedolizumab for ulcerative colitis and Crohn’s disease. Gut.

[133] Meserve J., Aniwan S., Koliani-Pace J.L., Shashi P., Weiss A., Faleck D., Winters A., Chablaney S., Kochhar G., Boland B.S. (2019). Retrospective Analysis of Safety of Vedolizumab in Patients with Inflammatory Bowel Diseases. Clin. Gastroenterol. Hepatol..

[134] Amiot A., Serrero M., Peyrin-Biroulet L., Filippi J., Pariente B., Roblin X., Buisson A., Stefanescu C., Trang-Poisson C., Altwegg R. (2019). Three-year effectiveness and safety of vedolizumab therapy for inflammatory bowel disease: A prospective multi-centre cohort study. Aliment. Pharmacol. Ther..

[135] Card T., Ungaro R., Bhayat F., Blake A., Hantsbarger G., Travis S. (2020). Vedolizumab use is not associated with increased malignancy incidence: GEMINI LTS study results and post-marketing data. Aliment. Pharmacol. Ther..

[136] Sands B.E., Sandborn W.J., Panaccione R., O’Brien C.D., Zhang H., Johanns J., Adedokun O.J., Li K., Peyrin-Biroulet L., Van Assche G. (2019). Ustekinumab as Induction and Maintenance Therapy for Ulcerative Colitis. N. Engl. J. Med..

[137] Ochsenkühn T., Tillack C., Szokodi D., Janelidze S., Schnitzler F. (2020). Clinical outcomes with ustekinumab as rescue treatment in therapy-refractory or therapy-intolerant ulcerative colitis. United Eur. Gastroenterol. J..

[138] Chaparro M., Garre A., Iborra M., Sierra-Ausín M., Barreiro-de Acosta M., Fernández-Clotet A., de Castro L., Boscá-Watts M., Casanova M.J., López-García A. (2021). Effectiveness and Safety of Ustekinumab in Ulcerative Colitis: Real-world Evidence from the ENEIDA Registry. J. Crohn’s Colitis.

[139] Fumery M., Filippi J., Abitbol V., Biron A., Laharie D., Serrero M., Altwegg R., Bouhnik Y., Peyrin-Biroulet L., Gilletta C. (2021). Effectiveness and safety of ustekinumab maintenance therapy in 103 patients with ulcerative colitis: A GETAID cohort study. Aliment. Pharmacol. Ther..

[140] Wils P., Bouhnik Y., Michetti P., Flourie B., Brixi H., Bourrier A., Allez M., Duclos B., Serrero M., Buisson A. (2018). Long-term efficacy and safety of ustekinumab in 122 refractory Crohn’s disease patients: A multicentre experience. Aliment. Pharmacol. Ther..

[141] Xeljanz. https://www.ema.europa.eu/en/medicines/human/EPAR/xeljanz#authorisation-details-section.

[142] Panés J., Vermeire S., Lindsay J.O., Sands B.E., Su C., Friedman G., Zhang H., Yarlas A., Bayliss M., Maher S. (2019). Tofacitinib in Patients with Ulcerative Colitis: Health-Related Quality of Life in Phase 3 Randomised Controlled Induction and Maintenance Studies. J. Crohns Colitis.

[143] Sandborn W.J., Su C., Sands B.E., D’Haens G.R., Vermeire S., Schreiber S., Danese S., Feagan B.G., Reinisch W., Niezychowski W. (2017). Tofacitinib as Induction and Maintenance Therapy for Ulcerative Colitis. N. Engl. J. Med..

[144] Sands B.E., Armuzzi A., Marshall J.K., Lindsay J.O., Sandborn W.J., Danese S., Panés J., Bressler B., Colombel J.F., Lawendy N. (2020). Efficacy and safety of tofacitinib dose de-escalation and dose escalation for patients with ulcerative colitis: Results from OCTAVE Open. Aliment. Pharmacol. Ther..

[145] Sandborn W.J., Peyrin-Biroulet L., Quirk D., Wang W., Nduaka C.I., Mukherjee A., Su C., Sands B.E. (2020). Efficacy and Safety of Extended Induction with Tofacitinib for the Treatment of Ulcerative Colitis. Clin. Gastroenterol. Hepatol..

[146] Sandborn W.J., Ghosh S., Panes J., Vranic I., Su C., Rousell S., Niezychowski W., for the Study A3921063 Investigators (2012). Tofacitinib, an oral Janus kinase inhibitor, in active ulcerative colitis. N. Engl. J. Med..

[147] Olivera P.A., Lasa J.S., Bonovas S., Danese S., Peyrin-Biroulet L. (2020). Safety of Janus Kinase Inhibitors in Patients with Inflammatory Bowel Diseases or Other Immune-mediated Diseases: A Systematic Review and Meta-Analysis. Gastroenterology.

[148] Sandborn W.J., Panés J., D’Haens G.R., Sands B.E., Su C., Moscariello M., Jones T., Pedersen R., Friedman G.S., Lawendy N. (2019). Safety of Tofacitinib for Treatment of Ulcerative Colitis, Based on 4.4 Years of Data from Global Clinical Trials. Clin. Gastroenterol. Hepatol..

[149] FDA Approves Boxed Warning About Increased Risk of Blood Clots and Death with Higher Dose of Arthritis and Ulcerative Colitis Medicine Tofacitinib (Xeljanz, Xeljanz XR). https://www.fda.gov/drugs/drug-safety-and-availability/fda-approves-boxed-warning-about-increased-risk-blood-clots-and-death-higher-dose-arthritis-and.

[150] Shah S.C., Colombel J.F., Sands B.E., Narula N. (2016). Mucosal Healing Is Associated with Improved Long-term Outcomes of Patients with Ulcerative Colitis: A Systematic Review and Meta-analysis. Clin. Gastroenterol. Hepatol..

[151] Cholapranee A., Hazlewood G.S., Kaplan G.G., Peyrin-Biroulet L., Ananthakrishnan A.N. (2017). Systematic review with meta-analysis: Comparative efficacy of biologics for induction and maintenance of mucosal healing in Crohn’s disease and ulcerative colitis controlled trials. Aliment. Pharmacol. Ther..

[152] Vulliemoz M., Brand S., Juillerat P., Mottet C., Ben-Horin S., Michetti P., on behalf of Swiss IBDnet, an official working group of the Swiss Society of Gastroenterology (2020). TNF-Alpha Blockers in Inflammatory Bowel Diseases: Practical Recommendations and a User’s Guide: An Update. Digestion.

[153] Ma C., Beilman C.L., Huang V.W., Fedorak D.K., Wong K., Kroeker K.I., Dieleman L.A., Halloran B.P., Fedorak R.N. (2016). Similar Clinical and Surgical Outcomes Achieved with Early Compared to Late Anti-TNF Induction in Mild-to-Moderate Ulcerative Colitis: A Retrospective Cohort Study. Can. J. Gastroenterol. Hepatol..

[154] Murthy S.K., Greenberg G.R., Croitoru K., Nguyen G.C., Silverberg M.S., Steinhart A.H. (2015). Extent of Early Clinical Response to Infliximab Predicts Long-term Treatment Success in Active Ulcerative Colitis. Inflamm. Bowel Dis..

[155] Oussalah A., Evesque L., Laharie D., Roblin X., Boschetti G., Nancey S., Filippi J., Flourié B., Hebuterne X., Bigard M.A. (2010). A multicenter experience with infliximab for ulcerative colitis: Outcomes and predictors of response, optimization, colectomy, and hospitalization. Am. J. Gastroenterol..

[156] Mir S.A., Nagy-Szakal D., Smith E.O., Gilger M.A., Kellermayer R. (2014). Duration of disease may predict response to infliximab in pediatric ulcerative colitis. J. Clin. Gastroenterol..

[157] Solberg I.C., Lygren I., Jahnsen J., Aadland E., Høie O., Cvancarova M., Bernklev T., Henriksen M., Sauar J., Vatn M.H. (2009). Clinical course during the first 10 years of ulcerative colitis: Results from a population-based inception cohort (IBSEN Study). Scand. J. Gastroenterol..

[158] Doherty G., Katsanos K.H., Burisch J., Allez M., Papamichael K., Stallmach A., Mao R., Berset I.P., Gisbert J.P., Sebastian S. (2018). European Crohn’s and Colitis Organisation Topical Review on Treatment Withdrawal [‘Exit Strategies’] in Inflammatory Bowel Disease. J. Crohn’s Colitis.

[159] Mehta F. (2016). Report: Economic implications of inflammatory bowel disease and its management. Am. J. Manag. Care.

[160] Severs M., Oldenburg B., van Bodegraven A.A., Siersema P.D., Mangen M.J., on behalf of the initiative of Crohn’s and Colitis (2017). The Economic Impact of the Introduction of Biosimilars in Inflammatory Bowel Disease. J. Crohns Colitis.

[161] Abraham B., Quigley E.M.M. (2020). Antibiotics and probiotics in inflammatory bowel disease: When to use them?. Frontline Gastroenterol..

[162] Biancone L., Michetti P., Travis S., Escher J.C., Moser G., Forbes A., Hoffmann J.C., Dignass A., Gionchetti P., Jantschek G. (2008). European evidence-based Consensus on the management of ulcerative colitis: Special situations. J. Crohns Colitis.

[163] Sáez-González E., Moret I., Alvarez-Sotomayor D., Díaz-Jaime F.C., Cerrillo E., Iborra M., Nos P., Beltrán B. (2017). Immunological Mechanisms of Adsorptive Cytapheresis in Inflammatory Bowel Disease. Dig. Dis. Sci..

[164] Hibi T., Sameshima Y., Sekiguchi Y., Hisatome Y., Maruyama F., Moriwaki K., Shima C., Saniabadi A.R., Matsumoto T. (2009). Treating ulcerative colitis by Adacolumn therapeutic leucocytapheresis: Clinical efficacy and safety based on surveillance of 656 patients in 53 centres in Japan. Dig. Liver Dis..

[165] Kruis W., Nguyen P., Morgenstern J. (2015). Granulocyte/Monocyte Adsorptive Apheresis in Moderate to Severe Ulcerative Colitis—Effective or Not?. Digestion.

[166] Yokoyama Y., Matsuoka K., Kobayashi T., Sawada K., Fujiyoshi T., Ando T., Ohnishi Y., Ishida T., Oka M., Yamada M. (2014). A large-scale, prospective, observational study of leukocytapheresis for ulcerative colitis: Treatment outcomes of 847 patients in clinical practice. J. Crohns Colitis.

[167] Kobayashi T., Matsuoka K., Yokoyama Y., Nakamura T., Ino T., Numata T., Shibata H., Aoki H., Matsuno Y., Hibi T. (2018). A multicenter, retrospective, observational study of the clinical outcomes and risk factors for relapse of ulcerative colitis at 1 year after leukocytapheresis. J. Gastroenterol..

[168] Paramsothy S., Paramsothy R., Rubin D.T., Kamm M.A., Kaakoush N.O., Mitchell H.M., Castaño-Rodríguez N. (2017). Faecal Microbiota Transplantation for Inflammatory Bowel Disease: A Systematic Review and Meta-analysis. J. Crohns Colitis.

[169] Øresland T., Bemelman W.A., Sampietro G.M., Spinelli A., Windsor A., Ferrante M., Marteau P., Zmora O., Kotze P.G., Espin-Basany E. (2015). European evidence based consensus on surgery for ulcerative colitis. J. Crohns Colitis.

[170] Choi C.R., Al Bakir I., Ding N.J., Lee G.H., Askari A., Warusavitarne J., Moorghen M., Humphries A., Ignjatovic-Wilson A., Thomas-Gibson S. (2019). Cumulative burden of inflammation predicts colorectal neoplasia risk in ulcerative colitis: A large single-centre study. Gut.

[171] Lopez A., Ford A.C., Colombel J.F., Reinisch W., Sandborn W.J., Peyrin-Biroulet L. (2015). Efficacy of tumour necrosis factor antagonists on remission, colectomy and hospitalisations in ulcerative colitis: Meta-analysis of placebo-controlled trials. Dig. Liver Dis..

[172] Barnes E.L., Jiang Y., Kappelman M.D., Long M.D., Sandler R.S., Kinlaw A.C., Herfarth H.H. (2020). Decreasing Colectomy Rate for Ulcerative Colitis in the United States Between 2007 and 2016: A Time Trend Analysis. Inflamm. Bowel Dis..

[173] Rutgeerts P.J., Fedorak R.N., Hommes D.W., Sturm A., Baumgart D.C., Bressler B., Schreiber S., Mansfield J.C., Williams M., Tang M. (2013). A randomised phase I study of etrolizumab (rhuMAb beta7) in moderate to severe ulcerative colitis. Gut.

[174] Vermeire S., O’Byrne S., Keir M., Williams M., Lu T.T., Mansfield J.C., Lamb C.A., Feagan B.G., Panes J., Salas A. (2014). Etrolizumab as induction therapy for ulcerative colitis: A randomised, controlled, phase 2 trial. Lancet.

[175] clinicaltrials.gov A Study of the Efficacy and Safety of Etrolizumab Treatment in Maintenance of Disease Remission in Ulcerative Colitis (UC) Participants Who Are Naive to Tumor Necrosis Factor (TNF) Inhibitors (LAUREL). https://clinicaltrials.gov/ct2/show/NCT02165215.

[176] Danese S., Colombel J.F., Lukas M., Gisbert J.P., D’Haens G., Hayee B., Panaccione R., Kim H.S., Reinisch W., Tyrrell H. (2022). Etrolizumab versus infliximab for the treatment of moderately to severely active ulcerative colitis (GARDENIA): A randomised, double-blind, double-dummy, phase 3 study. Lancet Gastroenterol. Hepatol..

[177] Feagan B.G., Panes J., Ferrante M., Kaser A., D’Haens G.R., Sandborn W.J., Louis E., Neurath M.F., Franchimont D., Dewit O. (2018). Risankizumab in patients with moderate to severe Crohn’s disease: An open-label extension study. Lancet Gastroenterol. Hepatol..

[178] clinicaltrials.gov A Study to Assess the Efficacy and Safety of Risankizumab in Participants with Ulcerative Colitis. https://www.clinicaltrials.gov/ct2/show/NCT03398135.

[179] Sandborn W.J., Ferrante M., Bhandari B.R., Berliba E., Feagan B.G., Hibi T., Tuttle J.L., Klekotka P., Friedrich S., Durante M. (2020). Efficacy and Safety of Mirikizumab in a Randomized Phase 2 Study of Patients with Ulcerative Colitis. Gastroenterology.

[180] clinicaltrials.gov A Study to Evaluate the Long-Term Efficacy and Safety of Mirikizumab in Participants with Moderately to Severely Active Ulcerative Colitis (LUCENT 3). https://clinicaltrials.gov/ct2/show/NCT03519945.

[181] Feagan B.G., Danese S., Loftus E.V., Vermeire S., Schreiber S., Ritter T., Fogel R., Mehta R., Nijhawan S., Kempinski R. (2021). Filgotinib as induction and maintenance therapy for ulcerative colitis (SELECTION): A phase 2b/3 double-blind, randomised, placebo-controlled trial. Lancet.

[182] Sandborn W.J., Ghosh S., Panes J., Schreiber S., D’Haens G., Tanida S., Siffledeen J., Enejosa J., Zhou W., Othman A.A. (2020). Efficacy of Upadacitinib in a Randomized Trial of Patients with Active Ulcerative Colitis. Gastroenterology.

[183] clinicaltrials.gov A Study of the Efficacy and Safety of Upadacitinib (ABT-494) in Participants with Moderately to Severely Active Ulcerative Colitis (U-Accomplish). https://clinicaltrials.gov/ct2/show/NCT03653026.

[184] Sandborn W.J., Feagan B.G., D’Haens G., Wolf D.C., Jovanovic I., Hanauer S.B., Ghosh S., Petersen A., Hua S.Y., Lee J.H. (2021). Ozanimod as Induction and Maintenance Therapy for Ulcerative Colitis. N. Engl. J. Med..

[185] Vermeire S., Chiorean M., Panes J., Peyrin-Biroulet L., Zhang J., Sands B.E., Lazin K., Klassen P., Naik S.U., Cabell C.H. (2021). Long-term Safety and Efficacy of Etrasimod for Ulcerative Colitis: Results from the Open-label Extension of the OASIS Study. J. Crohn’s Colitis.

[186] Herfarth H., Vavricka S.R. (2022). 5-Aminosalicylic Acid Chemoprevention in Inflammatory Bowel Diseases: Is It Necessary in the Age of Biologics and Small Molecules?. Inflamm. Intest. Dis..

[187] Vavricka S.R., Scharl M., Gubler M., Rogler G. (2014). Biologics for extraintestinal manifestations of IBD. Curr. Drug Targets.

